# Effectiveness of Tuina Therapy Combined With Yijinjing Exercise in the Treatment of Nonspecific Chronic Neck Pain: A Randomized Clinical Trial

**DOI:** 10.1001/jamanetworkopen.2022.46538

**Published:** 2022-12-13

**Authors:** Zi-ji Cheng, Shuai-pan Zhang, Yuan-jia Gu, Zi-ying Chen, Fang-fang Xie, Chong Guan, Min Fang, Fei Yao

**Affiliations:** 1 Tuina Department, Shanghai Municipal Hospital of Traditional Chinese Medicine, Shanghai, China; 2 School of Acupuncture-Moxibustion and Tuina, Shanghai University of Traditional Chinese Medicine, Shanghai, China; 3 Tuina Department, Yueyang Hospital of Integrated Traditional Chinese and Western Medicine, Shanghai University of Traditional Chinese Medicine, Shanghai, China

## Abstract

**Importance:**

Both tuina therapy and yijinjing exercise were beneficial to patients with nonspecific chronic neck pain, but the evidence for this combination is limited.

**Objective:**

To investigate the effectiveness of tuina therapy combined with yijinjing exercise compared with tuina therapy alone for patients with nonspecific chronic neck pain.

**Design, Setting, and Participants:**

A 12-week, open-label, analyst-blinded randomized clinical trial (8-week intervention plus 4-week observational follow-up) was conducted from September 7, 2020, to October 25, 2021. A total of 102 participants with nonspecific chronic neck pain were recruited, and data were analyzed from December 10, 2021, to March 26, 2022.

**Interventions:**

Participants in the tuina group or tuina combined with yijinjing group received 3 sessions of tuina therapy per week for 8 weeks, for a total of 24 sessions. Participants in the tuina combined with yijinjing group practiced yijinjing 3 times a week for 8 weeks, including an instructor-guided exercise at the hospital and 2 self-practice exercises at home.

**Main Outcomes and Measures:**

The primary outcome was change in visual analog scale (VAS) score from baseline to week 8. Secondary outcomes included Neck Disability Index scores, Self-rating Anxiety Scale scores, tissue hardness, and active range of motion.

**Results:**

This randomized clinical trial recruited 102 patients (mean [SD] age, 36.5 [4.9] years; 69 [67.6%] female) who were randomized to 2 groups. All 102 patients (100%) completed all the outcome measurements. The mean difference in VAS scores from baseline at week 8 for the tuina combined with yijinjing group was −5.4 (95% CI, −5.8 to −5.1). At week 8, the difference in VAS score was −1.2 (95% CI, −1.6 to −0.8; *P* < .001) between the tuina group and the tuina combined with yijinjing group. The effectiveness of tuina combined with yijinjing in treating nonspecific chronic neck pain remained at the 12-week follow-up.

**Conclusions and Relevance:**

In this randomized clinical trial, for patients with nonspecific chronic neck pain, tuina combined with yijinjing was more effective than tuina therapy alone in terms of pain, functional recovery, and anxiety at week 8, and effectiveness remained at week 12. A combination of tuina and yijinjing should be considered in the management of nonspecific chronic neck pain.

**Trial Registration:**

Chinese Clinical Trial Registry: ChiCTR2000036805

## Key Points

**Question:**

Is tuina combined with yijinjing more effective than tuina alone for nonspecific chronic neck pain?

**Findings:**

In this randomized clinical trial of 102 individuals with nonspecific chronic neck pain, the combined therapy had a statistically significant advantage in reducing pain at week 8 compared with tuina therapy alone. The effectiveness was still present at 12-week follow-up.

**Meaning:**

These findings suggest that a combination of tuina therapy and yijinjing exercise was more effective than tuina therapy alone in the treatment of patients with nonspecific chronic neck pain.

## Teaser

This randomized clinical trial compares tuina therapy combined with yijinjing exercise with tuina therapy alone for the treatment of patients with nonspecific chronic neck pain.

## Introduction

Neck pain is a common musculoskeletal disorder with a high prevalence worldwide. It is the fourth most common cause of disability in the US. The mean lifetime prevalence of neck pain is 48.5% (range, 14.2%-71%), the third highest in the US after diabetes and heart disease.^1^ Compared with low back pain, neck pain has not received enough attention.^2^ No specific pathology could explain the cause of neck pain (eg, nerve root compression). Patients with symptoms that persist for more than 3 months can be diagnosed as having nonspecific chronic neck pain (NCNP).^3^ In addition, NCNP is often associated with anatomical, psychological, social, and occupational factors. Anxiety and depression are also thought to be associated with higher levels of pain in patients with musculoskeletal pain.^4^

Because NCNP often occurs without any established pathologic process and cause, it is difficult to adopt precise treatment methods. Therefore, drugs, intra-articular injections, and surgery are often used as common treatments. However, the efficacy of these treatments is not guaranteed.^5,6^ Therefore, nondrug therapy has attracted more attention.^7^ Exercise is considered a treatment modality for pain relief. A previous systematic review found that multiple forms of exercise are beneficial to neck pain and disability.^8^ Complementary and alternative medicine, such as manual therapy, osteopathic therapy, and Qigong, have been used widely in treating NCNP.^9,10^

Tuina is a Chinese manual therapy that consists of 2 passive treatments. One is soft tissue manipulation, which consists of manual techniques, such as pressing, pushing, and kneading. The other is spinal manipulation, including high-velocity, low-amplitude thrust procedures or low-velocity, variable-amplitude mobilization.^11^ Systematic reviews have reported that tuina therapy can alleviate pain and relax stiff soft tissue for patients with NCNP.^12,13^ However, research on the direct physiologic mechanisms of tuina therapy for neck pain is relatively limited. Two previous basic studies on animal experiments showed that pain behavior of mice was improved after simulating massage manipulation, which was associated with reduced peripheral inflammation mediated by the mechanosensitive channel protein Piezo and senescence-related pathways.^14,15^ A recent study simulated the treatment process of tuina manipulation and found that the analgesic effect on a neuropathic pain model may be related to the inflammatory pathway regulated by noncoding RNA.^16^ Yijinjing is a type of traditional Chinese exercise that puts emphasis on the coordination of posture, meditation, and breathing. It is a moderately intense mind-body exercise that is easy to practice with few limitations.^17^ The results of a systematic study showed that physical and mental exercises, including yijinjing, are beneficial for the recovery of neck pain and disability.^18^ Two recent studies on the neuroimaging of patients with stroke after a yijinjing intervention reported that it can modulate brain neural network connections, which may be a central mechanism of its analgesia.^18,19^

Although tuina and yijinjing have been widely used in clinical treatment of NCNP in China, few high-level randomized clinical trials have been performed because of the limitations of traditional Chinese medicine research methods. In addition, nondrug therapy integrated into pain management is recommended by a clinical practice guideline.^20^ Thus, a 12-week, open-label, analyst-blinded randomized clinical trial was conducted to assess the effectiveness of tuina combined with yijinjing in treating NCNP. The results were measured by the patient-reported outcome visual analog scale (VAS) scores after the 8-week intervention. We hypothesized that tuina combined with yijinjing would play a better role in improving pain, disability, and anxiety.

## Methods

### Study Design

Full details of the trial have been published.^21^ A single-center, open-label, assessor-blinded randomized clinical trial was performed from September 7, 2020, to November 23, 2021, at Yueyang Hospital of Integrated Traditional Chinese and Western Medicine affiliated with Shanghai University of Traditional Chinese Medicine. All participants were recruited mainly through online social platforms, advertisements, and hospital posters. All participants provided written informed consent. The study was performed in accordance with the principles of the Declaration of Helsinki^22^ and approved by the Regional Ethics Review Committee of Yueyang Hospital. The reporting of the study complies with the Consolidated Standards of Reporting Trials (CONSORT) reporting guideline. The trial protocol can be found in Supplement 1.

### Eligibility Criteria

Participants with confirmed NCNP were eligible for the present study. Inclusion criteria were as follows: men or women aged 20 to 50 years whose VAS scores were 3 or higher and Neck Disability Index scores were 10 or higher, with chronic neck pain persisting for at least 3 months, with no history of shoulder and neck surgery, and with negative results on the neck distraction test, Spurling neck compression test, and Adson test. Exclusion criteria were as follows: specific disorders of the cervical spine, such as cervical radiculopathy or myelopathy; history of whiplash injury and/or head or neck injuries; being pregnant or lactating; neck pain radiating into the upper limb; history of severe trauma or tumor; having received clinical treatment for neck pain in the past 3 months; being unable to speak or write Chinese; adverse reactions to tuina and yijinjing; undergoing tuina or yijinjing in the past 3 months; and poor cooperation.

### Randomization, Allocation Concealment, and Blinding

After recruitment and baseline measurements, an independent office employee at the department generated the randomization list by a random-number generator (Strategic Applications Software, version 9.1.3; SAS Institute Inc). The randomization database was prepared at the same time. The random numbers were placed in sealed envelopes that had been numbered in order. The therapist opened envelopes sequentially in front of the patients and allocated the patients to the 2 groups in a 1:1 ratio randomly.

Except for the tuina therapist and yijinjing teacher, other researchers, including statisticians, outcome assessors, and data analysts, were blinded to group assignments. The tuina therapist and yijinjing teacher were not involved in the outcome assessment or data analysis.

### Interventions

Patients in both the tuina group and the tuina combined with yijinjing group received a total of 24 tuina treatment sessions (3 sessions per week for 8 consecutive weeks). Tuina was performed by a senior therapist who held a Traditional Chinese Medicine Practitioner Qualification License for more than 10 years. The intensity level of tuina was based on a physical examination and the therapist’s clinical experience after careful communication with each participant. A 3-step protocol, including soft tissue manipulation, clicking acupoint manipulation, and spinal manipulation, was performed by the tuina therapist to alleviate neck pain and restore neck function by relaxing the soft tissue of the neck and shoulder (eAppendix 1 in Supplement 2).

For patients in the tuina combined with yijinjing group, in addition to the 24 tuina sessions, a 5-step protocol of yijinjing was applied to improve the therapeutic effects. The 5 movements included the Third Aspect of Wei-tuo, taking away a star and changing the dipper for it, nine demons drawing their swords, bowing in salutation, and wagging the tail (eAppendix 2 in Supplement 2). Yijinjing was taught by a yijinjing teacher with 10 years of teaching experience. Yijinjing was practiced by the patients for 24 treatment sessions (3 sessions per week for 8 consecutive weeks). Each week, participants practiced yijinjing with the teacher once, then practiced it twice by themselves at home. A digital video disk about the movements was given to patients to review the movements in detail. The participants were required to upload videos and photographs of their own practices to the researchers. The videos and photographs were carefully examined by the yijinjing teacher. Some advice was given to the participants to help them practice yijinjing more effectively. All details about the intervention are shown in eAppendixes 1 and 2 in Supplement 2.

The tuina therapist and yijinjing teacher were trained for a week. They had passed a test to ensure consistency of study methods before participating in the trial.

### Outcomes

The primary outcome was the change in VAS score at the end of the intervention (week 8).^23,24^ The secondary outcomes included Neck Disability Index score, which provided a subjective assessment of patients’ function disability, consisting of 10 dimensions, such as pain and living standard.^25^ The Self-rating Anxiety Scale was used to assess anxiety level.^26^ The Chinese versions of the scales are all questionnaires that have obtained sufficient evidence of reliability and validity. Tissue hardness was measured by a digital algometer. The measuring point is placed between the C7 vertebra and the acromion at the middle point of the upper trapezius muscle. The active range of motion (AROM) was assessed with an electronic spine measuring device. Normal AROM of the neck was 30° to 45° flexion and extension, 30° to 45° left and right lateral flexion, and 60° to 80° left and right rotation. The measuring method is shown in eAppendix 1 in Supplement 2.

These outcomes were measured by blinded researchers. To avoid disclosing group assignments during the trial, patients were allowed only minimal conversation beyond what was necessary to measure the results at each measurement point. The researchers who used the digital algometer and the electronic spine measuring device participated in the 1-week training to understand how to use the instruments and passed relevant tests before participating in the trial. They were taught how to communicate with patients to avoid unnecessary conversation during the evaluation. We also collected outcome data at 12 weeks as an exploratory end point.

### Statistical Analysis

The sample size calculation was based on a previous study (n = 78) performed in 2016,^27^ which showed that the mean (SD) VAS scores were 5.5 (1.1) in the tuina group and 4.7 (1.3) in the tuina combined with yijinjing group after an 8-week intervention. Under the assumption that the superior effect was 1.3 of the difference in VAS score, with α = .05 and β = 0.1, the sample size was determined to be 84 patients (42 per group). With an assumed 20% dropout rate, 102 patients were recruited into this trial.

Data were analyzed from December 10 to March 26, 2022. Data analysis was based on the intention-to-treat principle. Descriptive analysis was used for the baseline characteristics of the patients in each group. All numerical data are presented as mean (SD). For quantitative data that did not conform to a normal distribution, data are expressed as median (IQR). In all analyses, statistical significance was accepted as a 2-tailed *P* < .05. For the primary outcome, VAS scores were assessed by using the linear mixed-effects model with the interaction effects of time and group. Participants who did not complete the study were treated as having no change from baseline at all times. A Bonferroni correction was used to account for multiple comparisons. Correlation analysis was conducted between the difference of the tissue hardness and the other outcome measures by the Pearson correlation analysis. All statistical analyses were conducted with SPSS software, version 24.0 (SPSS Inc).

## Results

### Participant Characteristics

**Figure 1.**
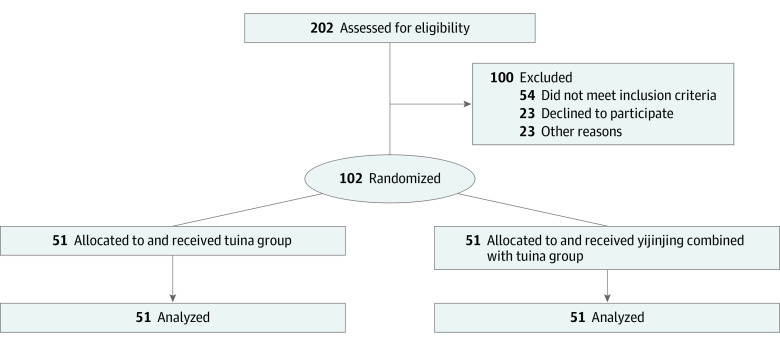
Study Flowchart

Of 202 potential participants who were screened, 102 patients (50.4%) with NCNP (mean [SD] age, 36.5 [4.9] years; 69 [67.6%] female and 33 [32.4%] male) who met the inclusion criteria were randomized to the tuina group (n = 51) or the tuina combined with yijinjing group (n = 51) (Table 1). All 102 patients (100%) completed all outcome measurements at week 8 (Figure 1).

**Table 1.** Baseline Characteristics of the Intention-to-Treat Population^a^

Characteristic	Tuina group (n = 51)	Tuina combined with yijinjing group (n = 51)
Age, mean (SD), y	36.2 (4.8)	36.8 (5.1)
Sex		
Male	15 (29.4)	18 (35.3)
Female	36 (70.6)	33 (64.7)
Height, mean (SD), y	166.0 (7.6)	166.1 (6.9)
Weight, mean (SD), y	61.9 (10.2)	61.8 (9.5)
VAS score, median (IQR)^b^	7 (6-8)	7 (6-8)
NDI score, mean (SD)^c^	26.4 (2.8)	26.4 (2.6)
SAS score, mean (SD)^d^	60.7 (4.7)	60.4 (4.0)
Tissue hardness, mean (SD), %^e^		
Tissue hardness of left upper trapezius muscle	76.3 (2.0)	75.9 (1.8)
Tissue hardness of right upper trapezius muscle	78.3 (2.1)	78.4 (2.0)
Active range of motion, mean (SD), degrees^f^		
Flexion	23.3 (2.5)	23.2 (2.8)
Extension	24.6 (3.0)	24.7 (2.7)
Left lateral flexion	23.3 (2.3)	22.8 (2.3)
Right lateral flexion	25.1 (2.3)	25.3 (2.4)
Left rotation	51.4 (2.5)	50.7 (2.9)
Right rotation	54.0 (3.2)	54.4 (3.1)

Abbreviations: NDI, Neck Disability Index; SAS, Self-rating Anxiety Scale; VAS, visual analog scale.

^a^ Data are presented as number (percentage) of participants unless otherwise indicated. No differences were found between groups for any characteristics at baseline.

^b^ On the VAS, higher scores indicate worse pain.

^c^ Scores range from 0 to 50, with higher scores indicating worse disability.

^d^ Scores range from 20 to 80, with higher scores indicating worse anxiety.

^e^ Measured by a digital algometer.

^f^ Measured by electronic spine measuring device.

### Efficacy

**Figure 2.**
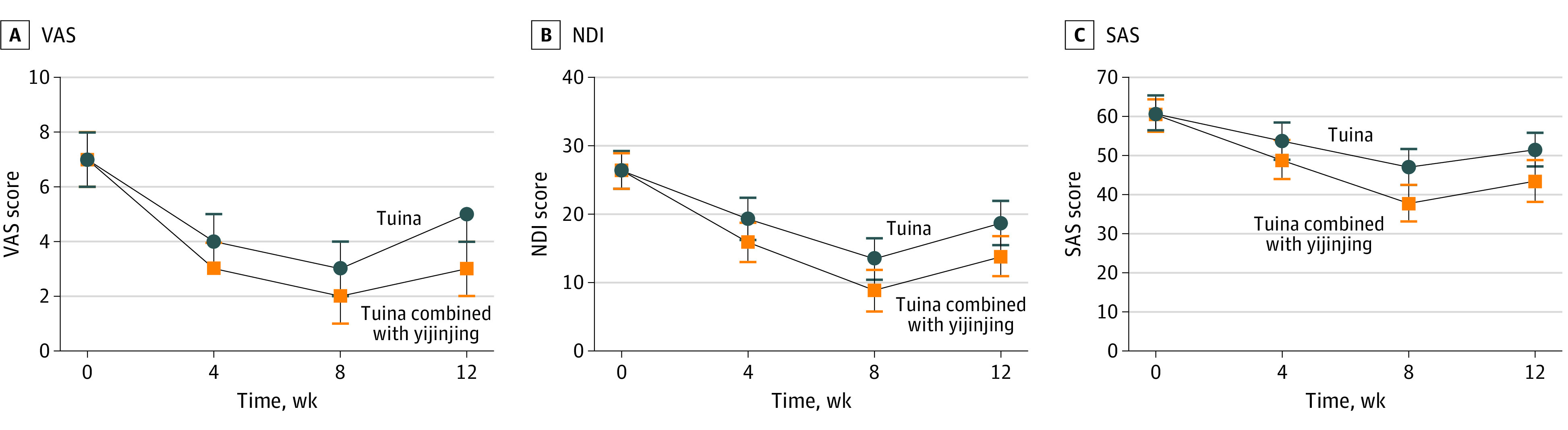
Changes in Outcomes Among Groups Over Time NDI indicates Neck Disability Index; SAS, Self-rated Anxiety Scale; and VAS, visual analog scale.

Table 2 gives the VAS scores of the 2 study groups; the results were analyzed to reveal changes from baseline (week 0) to 12-week follow-up (week 12). At the 8-week posttreatment assessment (primary end point), the tuina group had a mean reduction of −4.1 (95% CI, −4.4 to −3.8), and the tuina combined with yijinjing group had a mean reduction of −5.4 (95% CI, −5.8 to −5.1) in the VAS score from baseline. The tuina combined with yijinjing group showed a significant between-group difference in VAS score of −1.2 (95% CI, −1.6 to −0.8; *P* < .001) compared with the tuina group after the 8-week intervention period. A comparison of the VAS scores in the 2 groups is shown in Figure 2.

**Table 2.** VAS Scores Among Study Participants

Time	VAS score, median (IQR)		Mean change in VAS score from baseline (95% CI)^a^		Tuina group vs yijinjing combined with tuina group		Group × time interaction	Time	Group
	Tuina group (n = 51)	Yijinjing combined with tuina group (n = 51)	Tuina group	Yijinjing combined with Tuina group	Difference (95% CI)	*P* value^b^			
8 wk	3 (2 to 4)	2 (1 to 2)	–4.1 (–4.4 to –3.8)	–5.4 (–5.8 to –5.1)	–1.2 (–1.6 to –0.8)	<.001	χ^2^ = 58.9	χ^2^ = 1958.4	χ^2^ = 30.5
Baseline	7 (6 to 8)	7 (6 to 8)	NA	NA	NA	NA			
4 wk	4 (4 to 5)	3 (3 to 4)	–2.5 (–2.7 to –2.2)	–3.47 (–3.7 to –3.2)	–0.9 (–1.3 to –0.5)	<.001	*P* < .001	*P* < .001	*P* < .001
12 wk	5 (4 to 5)	3 (2 to 4)	–2.2 (–2.6 to –1.9)	–3.94 (–4.4 to –3.6)	–1.6 (–2.0 to –1.2)	<.001			

Abbreviations: NA, not applicable; VAS, visual analog scale.

^a^ On the VAS, higher scores indicate worse pain.

^b^ Compared using Bonferroni correction.

The secondary outcomes are given in Table 3. In the time point analysis, at the primary end point of week 8, compared with the tuina group, the tuina combined with yijinjing group showed significantly superior effectiveness for all secondary outcomes (function, −4.5 [95% CI, −5.5 to −3.6]; anxiety, −9.2 [95% CI, −10.9 to −7.6]; tissue hardness, −3.8 [95% CI, −4.8 to −2.8] for left upper trapezius muscle and *–*3.7 [*–*4.7 to *–*2.8] for right upper trapezius muscle; and active range of motion, 3.8° [95% CI, 2.9°-4.6°] for flexion, 4.2° [95% CI, 3.4°-5.0°] for extension, 3.8° [95% CI, 2.7°-4.8°] for left lateral flexion, 3.5° [95% CI, 2.7°-4.3°] for right lateral flexion, 6.6° [95% CI, 5.5°-7.6°] for left rotation, and 4.4° [95% CI, 3.4°-5.3°]) for right rotation.

**Table 3.** Secondary Outcomes at 4, 8, and 12 Weeks

Outcome	Mean (SD)		Mean change in NDI score from baseline (95% CI)		Tuina group vs yijinjing combined with tuina group		Group × time interaction	Time	Group
	Tuina group (n = 51)	Yijinjing combined with tuina group (n = 51)	Tuina group	Yijinjing combined with tuina group	Difference (95% CI)	*P* value^a^			
NDI^b^									
8 wk	13.53 (3.1)	8.89 (3.0)	*–*12.9 (*–*13.7 to *–*12.2)	*–*17.4 (*–*18.1 to *–*16.7)	*–*4.5 (*–*5.5 to *–*3.6)	<.001	*F* = 36.5	*F* = 1208.8	*F* = 43.2
Baseline	26.45 (2.8)	26.35 (2.6)	NA	NA	NA	NA			
4 wk	19.33 (3.1)	15.90 (2.9)	*–*7.1 (*–*7.6 to *–*6.6)	*–*10.5 (*–*11.1 to *–*9.8)	*–*3.4 (*–* 4.4 to *–*2.4)	<.001	*P* < .001	*P* < .001	*P* < .001
12 wk	18.76 (3.2)	13.86 (2.9)	*–*7.7 (*–*8.5 to *–*6.8)	*–*12.5 (*–*13.3 to *–*11.7)	*–*4.9 (*–*6.0 to *–*3.8)	<.001			
SAS^c^									
8 wk	47.04 (4.7)	37.80 (4.6)	*–*13.7 (*–*14.5 to *–*12.9)	*–*22.6 (*–*23.8 to *–*21.5)	*–*9.2 (*–* 10.9 to *–*7.6)	<.001	*F* = 72.1	*F* = 1062.5	*F* = 46.7
Baseline	60.73 (4.7)	60.43 (4.0)	NA	NA	NA	NA			
4 wk	53.75 (4.8)	48.78 (4.8)	*–*7.0 (*–*7.5 to *–*6.5)	*–*11.7 (*–*12.4 to *–*10.9)	*–*5.0 (*–*6.9 to *–*3.1)	<.001	*P* < .001	*P* < .001	*P* < .001
12 wk	51.55 (4.3)	43.49 (5.3)	*–*9.2 (*–*10.0 to *–*8.3)	*–*16.9 (*–*18.4 to *–*15.5)	*–*8.1 (*–*9.8 to *–*6.4)	<.001			
Tissue hardness of left upper trapezius muscle (%)^d^									
8 wk	67.60 (2.3)	63.77 (2.3)	*–*8.7 (*–*9.3 to *–*8.2)	*–*12.2 (*–*12.8 to *–*12.5)	*–*3.8 (*–*4.8 to *–*2.8)	<.001	*F* = 46.6	*F* = 1201.1	*F* = 61.5
Baseline	76.33 (2.0)	75.94 (1.8)	NA	NA	NA	NA			
4 wk	72.00 (2.0)	69.42 (2.0)	*–*4.3 (*–*4.6 to *–*4.0)	*–*6.5 (*–*7.0 to *–*6.1)	*–*2.6 (*–*3.5 to *–*1.7)	<.001	*P* < .001	*P* < .001	*P* < .001
12 wk	72.91 (1.8)	68.85 (2.1)	*–*3.4 (*–*4.0 to *–*2.9)	*–*7.1 (*–*7.6 to *–*6.6)	*–*4.1 (*–*5.0 to *–*3.2)	<.001			
Tissue hardness of right upper trapezius muscle, %^d^									
8 wk	67.60 (2.3)	63.77 (2.3)	*–*4.3 (*–*4.6 to *–*4.0)	*–*12.2 (*–*12.8 to *–*12.7)	*–*3.7 (*–*4.7 to *–*2.8)	<.001	*F* = 50.3	*F* = 1158.2	*F* = 40.7
Baseline	76.33 (2.0)	75.94 (1.8)	NA	NA	NA	NA			
4 wk	72.00 (2.0)	69.42 (2.0)	*–*4.2 (*–*4.6 to *–*8.2)	*–*6.8 (*–*6.2 to *–*7.2)	*–*2.3 (*–*3.2 to *–*1.3)	<.001	*P* < .001	*P* < .001	*P* < .001
12 wk	72.91 (1.8)	68.85 (2.2)	*–*3.4 (*–*3.9 to *–*2.9)	*–*6.9 (*–*7.4 to *–*6.4)	*–*2.5 (*–*4.2 to *–*3.4)	<.001			
Flexion, degree^e^									
8 wk	33.51 (2.4)	37.25 (1.8)	9.6 (10.2 to 10.8)	14.1 (13.3 to 14.9)	3.8 (2.9 to 4.6)	<.001	*F* = 24.9	*F* = 723.0	*F* = 50.6
Baseline	23.33 (2.5)	23.16 (2.8)	NA	NA	NA	NA			
4 wk	28.65 (2.5)	31.71 (2.5)	5.3 (4.8 to 8.8)	7.7 (8.6 to 9.4)	3.1 (2.1 to 4.0)	<.001	*P* < .001	*P* < .001	*P* < .001
12 wk	27.57 (2.4)	31.16 (2.3)	4.2 (3.6 to 4.9)	8.0 (7.0 to 9.0)	3.6 (2.7 to 4.5)	<.001			
Extension, degree^e^									
8 wk	34.75 (2.5)	38.92 (1.9)	9.5 (10.2 to 10.9)	14.2 (13.4 to 15.0)	4.2 (3.4 to 5.0)	<.001	*F* = 25.4	*F* = 703.3	*F* = 58.7
Baseline	24.55 (3.0)	24.73 (2.7)	NA	NA	NA	NA			
4 wk	30.02 (2.8)	33.10 (2.6)	5.5 (4.9 to 6.0)	8.4 (7.6 to 9.2)	3.1 (2.2 to 4.1)	<.001	*P* < .001	*P* < .001	*P* < .001
12 wk	28.69 (2.4)	32.90 (2.4)	4.1 (3.8 to 4.8)	8.2 (7.3 to 9.1)	4.2 (3.2 to 5.1)	<.001			
Left lateral flexion, degree^e^									
8 wk	32.78 (2.2)	36.55 (2.6)	9.5 (8.9 to 10.1)	13.8 (13.0 to 14.6)	3.8 (2.7 to 4.8)	<.001	*F* = 44.9	*F* = 900.9	*F* = 45.3
Baseline	23.29 (2.3)	22.75 (2.3)	NA	NA	NA	NA			
4 wk	28.41 (2.5)	30.88 (2.1)	5.1 (4.6 to 5.6)	8.1 (7.5 to 8.8)	2.5 (1.5 to 3.4)	<.001	*P* < .001	*P* < .001	*P* < .001
12 wk	26.65 (2.1)	30.80 (2.3)	2.8 (3.6 to 3.9)	8.06 (7.2 to 8.9)	4.2 (3.2 to 5.1)	<.001			
Right lateral flexion, degree^e^									
8 wk	35.53 (2.4)	39.04 (2.1)	10.4 (9.8 to 11.0)	13.7 (12.9 to 14.5)	3.5 (2.7 to 4.3)	<.001	*F* = 31.3	*F* = 891.0	*F* = 61.6
Baseline	25.14 (2.2)	25.33 (2.4)	NA	NA	NA	NA			
4 wk	31.02 (2.6)	34.18 (2.3)	5.9 (5.4 to 6.4)	8.8 (8.2 to 9.5)	3.2 (2.3 to 4.0)	<.001	*P* < .001	*P* < .001	*P* < .001
12 wk	29.02 (2.1)	33.55 (2.6)	3.9 (3.3 to 4.5)	8.2 (7.4 to 9.1)	4.5 (3.6 to 5.5)	<.001			
Left rotation, degree^e^									
8 wk	62.04 (2.3)	68.59 (2.4)	9.6 (10.2 to 10.8)	17.86 (16.9 to 18.8)	6.6 (5.5 to 7.6)	<.001	*F* = 85.9	*F* = 884.5	*F* = 170.8
Baseline	56.25 (2.6)	50.73 (2.9)	NA	NA	NA	NA			
4 wk	62.04 (2.3)	62.33 (2.1)	4.8 (4.1 to 5.5)	11.6 (10.9 to 12.4)	6.1 (5.1 to 7.1)	<.001	*P* < .001	*P* < .001	*P* < .001
12 wk	55.61 (2.8)	62.53 (2.3)	4.2 (3.3 to 5.0)	11.8 (10.9 to 12.7)	6.9 (5.9 to 8.0)	<.001			
Right rotation, degree^e^									
8 wk	67.80 (2.6)	72.16 (2.3)	13.0 (13.8 to 14.5)	17.8 (16.9 to 18.7)	4.4 (3.4 to 5.3)	<.001	*F* = 38.6	*F* = 1098.9	*F* = 84.5
Baseline	54.04 (3.1)	54.39 (3.1)	NA	NA	NA	NA			
4 wk	61.22 (2.7)	65.88 (2.5)	6.6 (7.2 to 7.7)	11.5 (10.6 to 12.4)	4.7 (3.6 to 5.8)	<.001	*P* < .001	*P* < .001	*P* < .001
12 wk	60.08 (2.6)	66.06 (2.7)	6.0 (5.1 to 7.0)	11.7 (10.7 to 12.7)	6.0 (4.9 to 7.1)	<.001			

Abbreviations: NA, not applicable; NDI, Neck Disability Index; SAS, Self-rating Anxiety Scale.

^a^ Calculated by using Bonferroni correction.

^b^ Scores range from 0 to 50, with higher scores indicating worse disability.

^c^ Scores range from 20 to 80, with higher scores indicating worse anxiety.

^d^ Measured by digital algometer.

^e^ Measured by electronic spine measuring device.

At the 12-week follow-up, the tuina combined with yijinjing group continued to show superior effectiveness for the treatment of NCNP (VAS score, −1.6 [95% CI, −2.0 to −1.2]; NDI score, −4.9 [95% CI, −6.0 to −3.8]; Self-rating Anxiety Scale score, −8.1 [95% CI, −9.8 to −6.4]; tissue hardness, −4.1 [95% CI, –5.0 to −3.2] on the left and –2.5 [95% CI, −4.2 to −3.4] on the right; and active range of motion, 6.0° [95% CI, 2.7°-4.5°] for flexion, 4.2° [95% CI, 3.2°-5.1°] for extension, 4.2° [95 CI, 3.2°-5.1°] for left lateral flexion, 4.5° [95% CI, 3.6°-5.5°] for right lateral flexion, 6.9° [95% CI, 5.9°-8.0°] for left rotation, and 6.0° [95% CI, 4.9°-7.1°] for right rotation). There were significant within-group differences in outcomes at weeks 4, 8, and 12. No adverse events occurred throughout the trial.

### Result of Correlation Analysis

Correlation analysis was conducted for the difference of the tissue hardness of the left and right upper trapezius muscle and the difference of scales and AROM before (week 0) and after intervention (week 8) in the tuina combined with yijinjing group. The difference in the tissue hardness of the left upper trapezius muscle was negatively correlated with the difference in flexion (*r* = −0.378; *P* = .006) and right rotation (*r* = −0.456; *P* = .001). The difference in the tissue hardness of left upper trapezius muscle was positively correlated with the difference in the tissue hardness of right upper trapezius muscle (*r* = 0.574; *P* < .001). The difference in the tissue hardness of the right upper trapezius muscle was negatively correlated with the difference in flexion (*r* = −0.517; *P* < .001) and right rotation (*r* = −0.497; *P* < .001). All outcomes are shown in eAppendix 3 in Supplement 2.

## Discussion

Nonspecific chronic neck pain is a clinical syndrome with high morbidity and recurrence rates, and complementary and alternative comprehensive therapies are recommended for its management. As traditional Chinese physical therapies, tuina and yijinjing are widely used in neck pain,^28,29^ which may explain the high patient adherence in this randomized clinical trial, with no dropout cases.

Compared with tuina, tuina combined with yijinjing was associated with a significantly lower VAS score by the end of the intervention period at week 8, and the alleviation in pain intensity persisted during the 12-week follow-up. At the end of the intervention, the patients in the tuina combined with yijinjing group also had greater reduction in disability and anxiety symptoms and tissue hardness, and the AROM were greatly improved at the same time.

A previous meta-analysis^9^ reported that traditional Chinese exercises could significantly reduce pain intensity for patients with chronic low back pain and improve their functional level. Consistent with these results, our findings suggest that the improvement in pain and disability in the tuina combined with yijinjing group was not only statistically significant but was also clinically significant. A similar study previously investigated the effectiveness of manual therapy combined with exercise.^30^ In addition, yijinjing had a good effect on the anxiety level of patients with NCNP. Yijinjing combines deep breathing with physical movements, which may have an influence on negative emotions. Wang et al^31^ found that traditional Chinese exercises, such as yijinjing, can reduce negative emotions and improve quality of life in patients with chronic diseases. Because yijinjing is an exercise with lower energy metabolism, most patients are highly adherent to yijinjing and are willing to recommend this treatment to others.

Tuina and yijinjing have been used to treat NCNP in China for thousands of years. However, little evidence exists on tuina for patients with NCNP, especially combined with yijinjing. To our knowledge, this randomized clinical trial is the first study to demonstrate the efficacy of tuina combined with yijinjing for patients with NCNP.

In the current trial, the efficacy of tuina combined with yijinjing on soft tissue hardness and AROM can be clearly observed by the electronic spine measuring device and digital algometer. The use of tuina combined with yijinjing significantly relieved pain intensity, disability, and level of anxiety. The mechanism of yijinjing in the treatment of NCNP is unclear. This is probably because yijinjing is similar to tai chi in that it can improve aerobic capacity and regulate the balance between dynamic and static mechanics of the neck, thereby improving neck function, controlling posture, and relieving pain.^32^ This rigorously designed and conducted trial, which included a longer study period, experienced tuina therapists and yijinjing teacher, and well-designed yijinjing movement, provides essential clinical evidence for tuina combined with yijinjing as an alternative treatment for NCNP.

### Limitations

This study has several limitations. First, because of the features of physical therapy interventions, the therapist and patients could not be blinded during the study; hence, performance bias may be introduced. Second, because of the single-center design of the trial, recruitment was limited to only Chinese patients, which might limit the generalization of the findings. Third, more women than men were included in the study. This disparity may have introduced some bias and limited the validity of the data. The number of male patients should be increased in subsequent trials. Fourth, there are differences in the intensity of the exercises because of patients’ different levels of proficiency in yijinjing. Although we set strict regulatory rules, it was still difficult to guarantee whether the participants practiced yijinjing exactly as required. Fifth, stratified randomization and block randomization were not applied in this trial, which may have led to underrepresentation of the sample. Sixth, our collection of results spanned approximately 12 weeks (baseline, 4 weeks during the intervention, at the end of the intervention [8 weeks], and 4 weeks after the intervention [12 weeks]). This design is to capture the maximum possible treatment effect, assuming that most benefit occurred immediately after the intervention and 12-week follow-up. Longer-term results are more clinically meaningful and should be collected and analyzed in future studies. Seventh, no control group was used because tuina therapy is considered experimental, and whether yijinjing exercise alone has a good effect on NCNP needs to be verified in future studies.

## Conclusions

In this randomized clinical trial, patients with NCNP who received tuina combined with yijinjing showed greater improvement in terms of pain intensity, disability, and anxiety than those who underwent tuina therapy. Tuina combined with yijinjing can be recommended for routine use as supplemental therapy for pain control in patients with NCNP.

## Notes

Supplement 1.
Trial Protocol

Supplement 2. **eAppendix 1.** Tuina Protocol **eAppendix 2.** Yijinjing Protocol **eAppendix 3.** Correlation Analysis

Supplement 3.
Data Sharing Statement
