# Supplementary material for: Effectiveness of Tuina Therapy Combined With Yijinjing Exercise in the Treatment of Nonspecific Chronic Neck Pain: A Randomized Clinical Trial
Source: JAMA Netw Open. 2022 Dec 13;5(12):e2246538. doi: 10.1001/jamanetworkopen.2022.46538 (PMC9856335; doi:10.1001/jamanetworkopen.2022.46538)
Supplement: Supplement 1. — Trial Protocol [file jamanetwopen-e2246538-s001.pdf]

Study Protocol..2

Study Protocol

Efficacy of Tuina combined with Yijinjing for patients with nonspecific chronic neck pain: a randomized controlled trial

Shanghai Municipal Hospital of Traditional Chinese Medicine
School of Acupuncture-Moxibustion and Tuina, Shanghai University of Traditional Chinese Medicine
Yueyang Hospital of Integrated Traditional Chinese and Western Medicine Affiliated to Shanghai University of Traditional Chinese Medicine

1. Background

Non-specific chronic neck pain (NCNP) is a serious health and public problem worldwide. Neck pain appears between the occipital condyle and C7 in the neck region [1]. Neck pain can be divided into specific neck pain and non-specific neck pain. Non-specific neck pain is also known as mechanical neck pain which is defined as simple neck pain without specific pathological changes and neurological impairments; it can be diagnosed as non-specific chronic neck pain if the symptoms persist more than 3 months [2]. Two-thirds of the adult population suffer from non-specific chronic neck pain [3]. According to the research, women are more likely to be affected than men [4]. The annual incidence of NCNP is increasing because of sedentary lifestyle and working conditions [5,6]. Patients' quality of life and efficiency of work decrease due to chronic pain, and high treatment expenses also cause a huge burden to society [7].

The mechanism of NCNP is still not clear. The researchers explained the mechanism from different aspects, such as the mechanical factors, EMG, and ROM. Altered muscle cross-sectional area, thickness, size, and activity of deep neck muscles have been mentioned repeatedly in the previous [8-10]. Rahnama et al. [11]showed the change of altered EMG activity and the atrophy of deep neck extensor in patients with NCNP which is thought to be the recurrence of NCNP. Barnsley et al. [12] demonstrated that limited ROM aggravates the tightness of the muscles surrounding the neck and joint adhesion which also leads to a decrease in biomechanical function of the neck, and this condition causes non-specific chronic neck pain. In addition, much attention has been paid to the scapular region. Dyskinesia of the scapula and misalignment of the scapula always follow with NCNP [13,14]. In addition, according to the "Bio-Psycho-Social" framework, repetitive and sedentary working conditions and postural abnormalities also contribute to NCNP [15]. Anxiety and depression associated with the existence of higher levels of pain in musculoskeletal pain conditions [16].

For most NCNP patients, first-line medication options always include analgesics like acetaminophen or non steroidal anti-inflammatory drugs (NSAIDs), but the effects of these drugs vary from person to person and they often do harm to the digestive, blood, urinary, and other systems because of the long-term use [17–19]. So, various complementary treatments have become more and more popular. Exercise therapy, ultrasound, acupuncture, electrical nerve stimulation, and manual therapy have also been used widely in treating NCNP [20]. However, poor standardization of experiments, small sample size, low-quality control, and insufficient objective index caused controversy about their efficacy.

Tuina therapy is also called Chinese massage [21]. Tuina has also been proved to be a feasible way to treat neck pain and has been widely used in China [22]. As an important part of Chinese traditional medicine, Tuina is a manual therapy with anatomical and physiological principles, putting emphasis on meridians and acupoints [23]. Tuina therapy mainly includes two parts: soft tissue manipulation and spinal manipulation. Soft tissue manipulation techniques include stroking, kneading, and drumming, which are also found in some Western massage techniques, and spinal

manipulation also combined with high-velocity low-amplitude thrust manipulation techniques [23]. Two systematic reviews had shown that Tuina therapy can reduce pain and muscle tension for patients with non-specific neck pain [24, 25]. Tuina therapy acts on soft tissue and connective tissue that may lead to local biochemical changes that regulate local blood circulation, improve muscle flexibility, enhance lymph movement, and loosen connective tissue adhesion, which may alternately improve local injury and inflammation of the reuptake mediator [26]. Chinese traditional exercise is also a kind of exercise therapy which puts attention on the coordination of posture, meditation, and breathing [27]. Exercise therapy has been proved to be good for non-specific chronic neck pain [28]. Yijinjing is an ancient Chinese traditional exercise which has been widely practiced for keeping fit and treating diseases. Yi means change, Jin refers to muscles and sinews, and Jing means methods, so Yijinjing means a series of exercises to change the muscle and sinews literally. Yijinjing is a low-intensity, noncompetitive, and non-impact exercise. TCM doctors often apply Yijinjing as a complementary therapy to NCNP. Yijinjing can reduce neck pain and disability, as well as reduce stress, anxiety, and depression by unique movements [29–31]. Based on previous researches, people can improve sub-health, reduce pain, and promote immune cell by practicing Yijinjing regularly [32, 33]. However, to confirm these findings, more studies with larger sample sizes, standardized trials, and adverse event reports are needed.

Studies have shown that a single treatment plan is not effective, so the combination therapy has received more attention and is recommended by related scholars [34, 35]. There is quite a little evidence for the efficacy of Tuina on non-specific chronic neck pain, especially when combined with Yijinjing. We hypothesize that Tuina and Yijinjing have beneficial effects on nonspecific chronic neck pain because that is a case with subacute and long-lasting neck pain [36]. We want to further explore whether Tuina combined with Yijinjing exercises can play a better role in pain, disability, and negative emotions. Thus, we designed a randomized controlled trial (RCT) to prove our hypothesis.This trial will provide a solid clinical foundation for the efficacy of Yijinjng combined with Tuina. It will be served as a prospective experiment as well.

2.Study objectives

This study is a randomized, evaluator and statisticianblinded, parallel-controlled, superiority trial. The purpose of this trial will be to assess the following: 1) Whether Tuina combined with Yijinjing is not inferior to Tuina regarding pain, disability, and negative emotions for patients with NCNP.

Specific primary objective

The specific primary objective is to determine the change in the visual analog scale (VAS) pain scores from baseline, 4 weeks during the intervention, at the end of the intervention(8 weeks), and 1 month after the intervention within and between the two groups.In particular, the change from baseline in VAS at week 8 was used as the primary outcome.

3.Methods/design

3.1Study design

This study is a single-center, randomized, and analyst-blinded controlled trial with two arms: Tuina group (control) and Yijinjng combined with Tuina group (intervention). The study protocol has been approved by the Regional Ethics Review Committee of Yueyang Hospital of Integrated Traditional Chinese and Western Medicine affiliated with Shanghai University of Traditional Chinese Medicine (project number: 2020-018). A total of 102 eligible NCNP patients will be recruited and assigned in a 1:1 ratio randomly. Written informed consent will be provided by all patients. Independent researchers who are blinded to the patient assignment will collect and analyze the outcome assessment and related data. The study design is illustrated in the flow chart in Fig1.

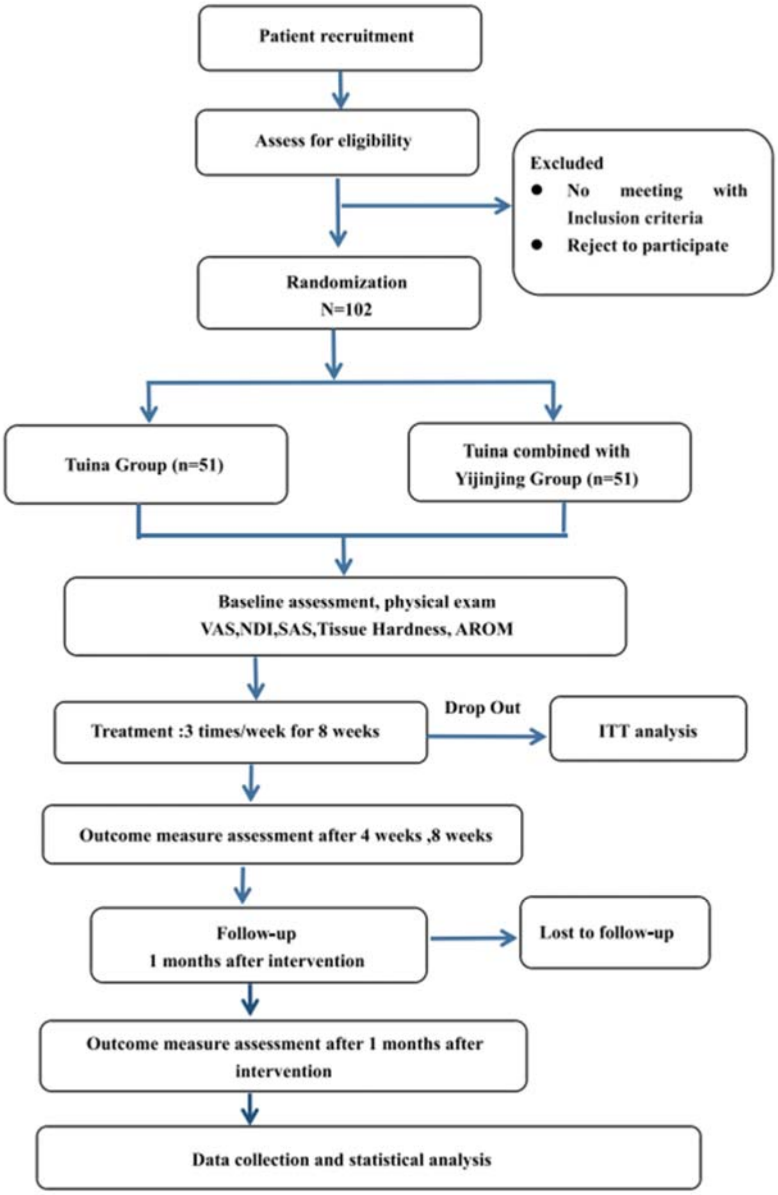

Fig1.Trial flowchart

3.2Participants and recruitment

Eligible participants include patients diagnosed with NCNP according to the base guideline for the chiropractic treatment of adults with neck pain which is summarized by GDC of Canada [37].Patients with non-specific neck pain for more than 3 months (no relief for more than 2 weeks) for the first time in the Tuina Department of Yueyang Hospital of Integrated Traditional Chinese and Western Medicine affiliated with Shanghai University of Traditional Chinese Medicine will be informed about this trial. If the patient expresses interest in this trial, a clinical trial communicator will make a contract with him/her immediately and make a brief introduction about the trial. If the patient decides to take part in this trial, he/she will have a face-to-face interview in a reception room of the Shanghai University of Traditional Chinese Medicine. Patients who meet the inclusion criteria will join the trial after they sign the informed consent form.

Inclusion criteria

Participants who meet all the following criteria can be enrolled:

1) Aged 20–50 ,2) Individuals from either sex ,3) Current neck pain (localized to the cervical or bilateral scapular region) ,4) Negative sign of neck distraction test, Spurling's neck compression test, and Adson's test ,5) Have neck pain symptoms of at least 3 months' duration ,6) Visual analog scale (VAS) $\geq$ 3 and Neck Disability Index (NDI) score $\geq$ 10 at recruitment time ,7) No previous shoulder or neck surgery and no accompanying shoulder problems 8) Willingness to participate.

Exclusion criteria

Participants meeting any of the following criteria will be excluded from this trial:

1) Specific disorders of the cervical spine, such as disk prolapse, spinal stenosis, postoperative conditions, cervical radiculopathy, or myelopathy ,2) History of whiplash injury and/or head/neck injuries ,3) Are pregnant or have had a recent delivery ,4) Response to prior treatment (a patient with neck pain radiating into the arm whose arm pain resolved with an injection or medication),5) History of severe trauma, spasmodic torticollis, frequent migraine, fibromyalgia, shoulder diseases, inflammatory rheumatic diseases, tumor, osteoporosis, psychiatric illness, and obvious spinal deformity or neurological disease6) No clinical treatment for neck pain in the past 3 months ,7) Unable to speak or write Chinese in order to complete the questionnaires ,8) Alcohol and drug abuse.9) Have an uncomfortable reaction to Tuina ,10) Subjects with regular practice of Yijinjing, Qigong, or Yoga in the past 3 months ,11) Poor cooperation.

3.3Randomization and allocation concealment

The randomization list will be generated by a random number generator (Strategic Applications Software, version 9.1.3; SAS Institute Inc., Cary, NC, USA). The random numbers will be placed in an opaque envelope which has been numbered in order. Before implementing random assignment, the research team will record the detailed information of each participant in the clinical center, including the new participant (name, date of birth, participant and center code, and date of inclusion) during reporting and preparation of a signed informed consent. The therapist will sequentially open the envelopes and allocate the participants accordingly. Eligible

participants will be randomly assigned to the experimental group and the control group according to 1:1 equal proportion rules after the baseline assessment.

3.4Blinding

Patients will be informed of the type of treatment that they will receive. The therapists will know the allocation so they should learn how to communicate with patients to ensure treatment blinding. In order to reduce the risk of bias, evaluators, data managers, and statisticians will be unaware of the group assignments in the result evaluation procedures and data analysis. The blinding procedure will be operated until the data are locked.

3.5Interventions

The Tuina protocol used in this trial is the same as those used in our previous studies [38, 39]. It includes soft tissue manipulation and spinal manipulation, such as rolling, pressing, and tapping. Yijinjng for NCNP patients was designed on the basis of the textbook which has been used for teaching students in the universities of TCM [40, 41].

Participants in the Tuina group or the Yijinjng combined with Tuina group will receive Tuina treatment 3 times a week for 8 weeks. The treatment room will be controlled at 23–25°to ensure that the participants feel comfortable. The participants will be asked to rest for 15 min before Tuina treatment. They will be advised to lie in the prone position during the treatment. The intensity level of Tuina is based on physical examination and the therapist's clinical experience, as well as after careful communication with each study participant. Tuina treatment will last for 25 min.

The participants who are in the Yijinjng combined with Tuina group will practice Yijinjing 3 times a week for 8 weeks. The patients will be assembled once a week for practicing Yijinjing together. The Yijinjing teacher will teach them how to practice Yijinjing. The teacher will also correct the wrong movement of patients. The patients will practice Yijnjing another two times per week at home. A digital video disk about the movements of Yijinjing in this trial will be provided to the participants. They can review the movements at home easily. The participants are asked to film themselves and sent it to the teacher by email or WeChat. The teacher will examine the participants'video carefully and give the patients some advice about practicing Yijinjing. Yijinjing treatment will last for 30 min.

Tuina group

In this arm of study, the Tuina therapist will administer a three-step protocol intended to alleviate neck pain and restore neck function by relaxing the soft tissue of the neck and shoulder. The specific protocol used is described below.

Step 1: Soft tissue manipulation

Patients are instructed by the therapist to lie in the prone position and to relax their mind and body naturally. Non-specific chronic neck pain conditions will be carefully examined by postural and palpatory assessment prior to treatment. The therapist will relax soft tissue and stiff muscles of the neck and shoulder by pressingkneading manipulation for 5 min. Then, the therapist will use his palms to roll the trapezius muscle gently so as to relax the back area for 5 min. The aim of this step is to resolve adhesion and increase general circulation.

Step 2: Clicking on the acupuncture point manipulation

The therapist will press and knead GB20, DU16, GB21, SJ14, and SI14 for 2 min each. This step is performed to unblock Qi stagnation and remove blood stasis by separating adherent fascicles. The amount of force used is determined by the patient's Deqi sensation, often described as a dull pain, heaviness, numbness, or soreness, and it is commonly regarded as an indicator of manipulation effectiveness in acupuncture and Tuina [42, 43].

Step 3: Spinal manipulation

The spinal manipulation will be used after the above two steps have relieved the tensions of the muscles and soft tissues. The patient will be instructed to sit in an orthopnea position in order to ensure the safety of manipulation. First, the therapist can exert a gentle torque to align the patient's neck area and use the shake method to relax this area. Then, the therapist will press one thumb on the deviated spinous process while another palm holds the lower jaw. Using gentle tractions and twisting of the neck, the therapist should hold this position for a moment and then made an abrupt pulling motion to advance the stretch by 5 to 10°.

Yijinjing combined with Tuina group

In this arm of study, the steps of Tuina treatment are the same as the Tuina group. The Yijinjing teacher will administer a five-step protocol intended to improve the therapeutic effects and adjust physical and mental conditions. The main movements of Yijinjing are shown in Fig. 2

Step 1: The third aspect of Wei-tuo ,Step 2: Taking away a star and changing the Dipper for it,Step 3: Nine demons drawing their swords,Step 4: Bowing in salutation,Step 5: Wagging the tail.

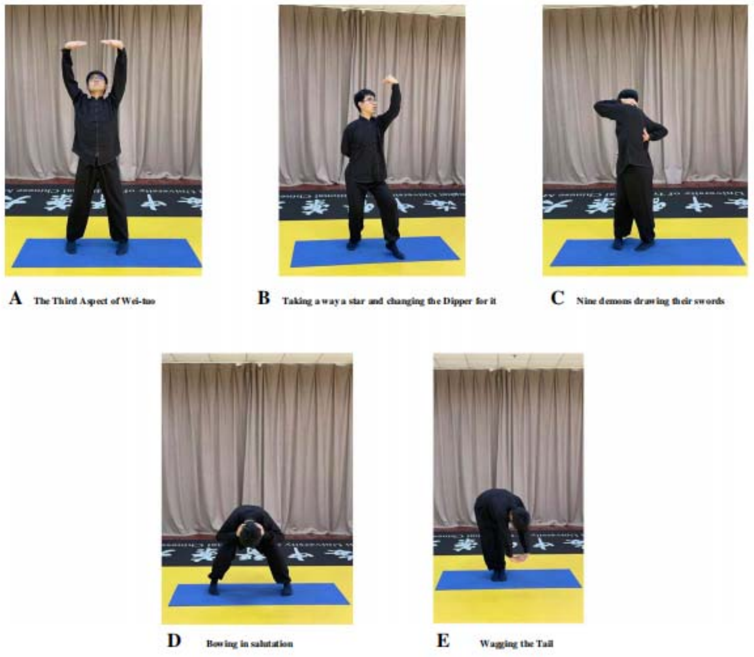

Fig2. The main movements of Yinjining

3.6Outcome measurements

Primary outcome measurement

VAS

The intensity of NCNP will be measured by a scale of 10-cm horizontal line visual analog scale (VAS) [44]. The patients will be asked “How much pain do you have this moment?”, then the patient will mark on the 10-cm horizontal line visual analog scale. Zero means “absence of pain,” while 10 represent “the worst pain.” VAS has been proved as a valid and reliable outcome measure for recording pain with ICC = 0.96 to 0.98 according to a previous study [45].

Secondary outcome measurements

NDI

The Neck disability index(NDI) will be used to measure the patients' limitations in everyday-life activities because of neck function[46]. It is is most commonly used as a self-reported questionnaire in neck pain.It contains 10 questions , each of which comprise 6 potential answers ranging from 0 (no disability) to 5 (full disability) and the total of the NDI score varies between 0 and 50 points.The total NDI scores less than 4 indicate no disability; 5-14, mild disability; 15-24, moderate disability; 25-34, severe disability;more than 35 points “complete disability.”

SAS

The anxiety level of NCNP patient will be measured by Zung self-rating anxiety scale(SAS)[47]. 20 questions are divided into 4 groups:cognitive, autonomic, motor, and central nervous system symptoms. Each section is scored on four levels of anxiety intensity from “1” not at all" to “4”very much" and with a sum score between 20and 80. A higher total score indicates a more severe anxiety level. The total raw scores range from 20 to 80. The raw score consequently needs to be converted to an “Anxiety Index”. The primary scores should be interpreted into Anxiety Index. The clinical interpretation of one's level of anxiety is as follows: 20-44 Normal Range; 45-59 Mild to Moderate.60-74 marked to severe lever;75-80 extreme Anxiety Level.

Tissue Hardness

Tissue hardness is measured by a digital algometer (OE-220, ITO, Tokyo, Japan) The measurement has been use to test the tissue hardness at previous study[48,49,50]. The measuring point is placed between C7 and acromion at the middle point of the upper trapezius muscle. The researcher put the meter on the measuring point perpendicularly and push the force slowly.When hearing the deep sound,the researcher should stop pushing and read the number. To standardize the speed of using this application, the researchers responsible for this measurement will practice one week before the study. They must explain the measurement by demonstrating at the the nar region of the hand. Three soft tissue measurements will be preformed at each point with an interval of 30 seconds between the two measurements,the mean of three measurements will be record.

Active range of motion (AROM)

Cervical active range of motion is measure by Spain ScanTM SH-105 (Ad-Or Medical Technologies Ltd, Israel). The validity and reproducibility of this measurement have been proved by many researchers[51,52]. SH-105 is composed of goniometer and a computer. The data will be transmitted by bluetooth. The patient

will be seated with a straight back leaning against the back of a chair. The goniometer will be placed on the centre of the cranial. The researcher switch the mode and long press the "start/end" button. When hearing the prompt sounds,the patient will be asked to flex forward to the limit. The patient will be instructed to stop at the point where pain symptoms preform. The research press the "Start/end" button again and the computer will automatically record the data. Each movement(flexion-extension, lateral flexion as well as rotation) will be measured in this way three time.The mean of each movement will be recorded.

Safety evaluation

The safety of patients will be monitored in every visit. Any adverse events (AES) will be evaluated by the researchers. The AES in trail such as changes in pain,syncope, vertigo,disability and etc. For any AES, no matter it is or not caused by intervention, the treatment will be stopped immediately. The patient should take any medical aids to alleviate symptoms. The adverse events should be report to the the relevant responsible unit and the ethics committee in time.

Follow-up

To evaluate the long-term efficacy and safety of the intervention,we will follow up the patients after treatments for 1 months. All the specific interventions and outcome measures assessment process are shown in Fig3.

Period	inclusion	Treatment		Follow-up
Assessment	baseline	First	Second	Third
Measure Point	0 week after inclusion	4 weeks after inclusion	8 weeks after inclusion	12 weeks after inclusion
inclusion confirmed	✓			
Informed consent	✓			
Physical exam	✓	✓	✓	✓
Disease history	✓			
Treatment history	✓			
Comorbidity	✓	✓	✓	✓
Current treatment	✓	✓	✓	✓
Pain condition and neck function assessment				
Visual analogue scale (VAS)	✓	✓	✓	✓
Neck Disability Index (NDI)	✓	✓	✓	✓
Self-Rating Anxiety Scale(SAS)	✓	✓	✓	✓
Tissue hardness	✓	✓	✓	✓
Active Range Of Motion(AROM)	✓	✓	✓	✓
Data collection and statistical analysis				
Adverse event		✓	✓	✓
Causes of dropout		✓	✓	✓
Safety analysis		✓	✓	✓
Compliance analysis		✓	✓	✓

Fig3. All interventions,measurements and measuring time points

3.7Sample size calculation

The following two hypotheses are related to the differences between the two groups.

H0:$\mu1-\mu2\leq\Delta$

H1:$\mu1-\mu2>\Delta$

where μ1 is the VAS score for treating 8 weeks in Tuina group, and μ2 is the VAS score for treating 8 weeks in the Tuina combined with Yijinjing group.According to a previous clinical study in China[53], the mean and standard deviation of VAS in Tuina group(n=34) after intervention was 5.5 and 1.1.The the mean and standard deviation of VAS in Tuina combined with neck exercise group(n=34) after intervention was 4.7 and 1.3.The following formula as used to calculate the sample size in this trial:

$$n = \frac{2(z_{\alpha/2} + z_{\beta})^2 \times \sigma^2}{(\mu_2 - \mu_1 - \Delta)^2}$$

$$n = \frac{2(0.025 + 0.2)^2 \times 1.3^2}{(4.7 - 5.5 - 0)^2} = 42$$

(α=0.025 β=0.2, superiority design,Δ=0)Considering a dropout rate of 20%, each group will require 51 cases. Therefore, a total of 102 participants should be recruited for this randomized controlled trial (RCT).

3.8Statistical analysis

All statistical analyses will be performed with SPSS Software (SPSS, version 24.0, SPSS Inc., Chicago, IL, USA) by statisticians who are independent of the research team and blinded to the group allocation. Data analysis will be based on the intention-to-treat (ITT) principles.The statistical significance was accepted for values of $p<0.05$. Participants who fail to complete the study will be treated as having no change from baseline at all times. Descriptive statistics will be used to compare demographic and baseline information and evaluate the credibility of the groups. The normality of data will be tested by Kolmogorov–Smirnov test. Parametric statistics (Tukey test) or non-parametric statistics (Wilcoxon rank sum test) will be used for the within- and between-group according to the results of homogeneity and normality analysis. If the data does not conform to a normal distribution,a covariance analysis will be used. The efficacy will be measured at four time points. A repeated measures analysis of variance will be conducted to analyze dependent variables (from baseline and follow-up). Bonferroni and Dunn tests will be used for multiple comparisons. The intra-group comparison (comparison between baseline and follow-up) will be tested by two-sided paired t test.The difference of categorical variables(VAS,NDI,SAS,PPT,AROM) and adverse effects between groups will be analyzed by using Chi-square test or Fisher's exact test. All numerical data will be presented as the mean±SD and categorical variables will be described with percentages (%). If it is necessary, post hoc analyses will be performed. The confidence interval will be established at 95% and the significance level at 0.05.

3.9Ethical considerations

The study collects data from patients with NCNP. The inform consent are required from the patients. Patients can terminate participation at any time.The results of the trial will be shown in tables and figures only and no individual will be identified. All data collected from this study can only be used for this research. All members of the research team have ethical principles of confidentiality.In addition, we will try our best to deal with ethical issues which arise during the study. We estimate that the benefits of the study far outweigh any possible risks.The trial has been approved by the ethics committee of Yueyang Hospital of Integrated Traditional Chinese and Western Medicine, which is affiliated with Shanghai University of Traditional Chinese Medicine(2020-018) and registered on ChiCTR(2000036805).

4.Discussion

Nonspecific chronic neck pain(NCNP) is a common and high prevalence musculoskeletal problem in the world. It can cause ADL ,work disability and economic cost and psychological stress[54,55]. Because of high recurrence rate, a scientific and reasonable intervention should be explored and promoted. It can not only relieve symptoms and reduce the burden of individual and society, but also decrease complication and improve the quality of life. Yijinjing and Tuina are important components of traditional Chinese medicine. They have been used thousands of years to keep people healthy. Quite a few people know the concrete operating procedure,advantages ,efficacy of such interventions. Therefore, the aim of this study is to investigate whether Yijinjing combined with Tuina has any superiority to than Tuina.

According to TCM, a healthy human body depends on the coordination of the internal organs and the harmony of qi and blood. The main reason of pain will be attribute to qi stagnation and blood stasis. In this study , relaxing manipulation,clicking on the acupuncture point manipulation and neck structural rectification are chosen to release adhesion and smooth joint movement so as to alleviate pain improve joint movement condition. By using these manipulations, the circulation of qi and blood will be promoted. Five movements of Yijnjing which are specifically for neck are chosen. These movements include neck flexion, rotation and lateral flexion. The features of Yijijing is the harmony of body, breath and mind. It puts emphasis on the unity of strength and meditation by using static postures and dynamic movements. It can circulate qi and blood, strengthen muscles and nourish tissues and organs and also arrest spasm[56].Previous study demonstrated that regular and long-term training of Yijinjing can raise the skeletal muscle strength and improve the motor function and ADL[57-58]. A clinical research reported that after 6 month Yijinjing multiple factors of depression and anxiety dropped significantly. It indicated that Yijinjing can improve patients' mental conditions[59] .

The present trail is a comparative efficacy of Tuina(control) and Yijinjing combined with Tuina (intervention) for the physical and mental symptoms of NCNP patient. We want to explore whether Yijinjing combined with Tuina is better than Tuina. We will evaluate three aspect of neck pain:pain,physical function and mental function. we will use validated scales and questionnaires and some measuring instrument to assess the clinical outcomes.

Pain is the most important symptom of neck pain, so visual analog scale(visual analog scale) will be used as the primary outcome.It. It can evaluate the intensity of neck pain. The secondary outcomes are NDI,which can evaluate the limitation of daily life because of NCNP. Cervical active range of motion (AROM) which can evaluate the neck active range of motion is measure by using a easy equipment.SAS ,which can evaluate the anxiety level of patients.Tissue Hardness will be measured by a tissue hardness meter and expressed in numbers.

To the best of our knowledge, no study has proved the efficacy of Yijinjing combined with Tuina for patients with NCNP.

High quality clinical data will be collected because of the rigorous experimental design. The efficacy of this specific intervention protocol for treating NCNP will be

evaluated by these data. We hope that this study will provide a solid foundation for the treatment of NCNP,as well as Tuina and Yijinjing research.

References

1.Gummesson C,Isacsson SO,Isacsson AH,et al. The transition of reported pain in different body regions--a one-year follow-up study. BMC Musculoskeletal Disorders, 2006,7:17.

2.López-de-Uralde-Villanueva I, Beltran-Alacreu H, Fernández-Carnero J, La Touche R. Pain management using a multimodal physiotherapy program including a biobehavioral approach for chronic nonspecific neck pain: a randomized controlled trial. Physiother Theory Pract. 2020;36(1):45-62.

3.Chiu TT, Leung AS. Neck pain in Hong Kong: a telephone survey on prevalence, consequences, and risk groups. Spine (Phila Pa 1976). 2006;31(16):E540-E544.

4.Lau HMC, Wing TT, Lam TH. The effectiveness of thoracic manipulation on patients with chronic mechanical neck pain – A randomized controlled trial. Man Ther .2011;16(2):141-147.

5.Feng B, Liang Q, Wang Y, Andersen LL, Szeto G. Prevalence of work-related musculoskeletal symptoms of the neck and upper extremity among dentists in China. BMJ Open. 2014;4(12):e006451.

6.Hogg-Johnson S, van der Velde G, Carroll LJ, et al. The burden and determinants of neck pain in the general population: results of the bone and joint decade 2000–2010 task force on neck pain and its associated disorders. J Manipulative Physiol Ther. 2009;32(2):S46–S60.

7.Haldeman S, Carroll L, Cassidy JD. Findings from the bone and joint decade 2000 to 2010 task force on neck pain and its associated disorders. J Occup Environ Med. 2010;52(4):424–427.

8.Wermeling M, Scherer M, Himmel W. GPs' experiences of managing non-specific neck pain--a qualitative study. Fam Pract. 2011;28(3):300–306.

9.Falla DL, Jull GA, Hodges PW. Patients with neck pain demonstrate reduced electromyographic activity of the deep cervical flexor muscles during performance of the craniocervical flexion test. Spine. 2004;29(19):2108–2114.

10.Kim JY, Kwag KI. Clinical effects of deep cervical flexor muscle activation in patients with chronic neck pain. J Phys Ther Sci. 2016;28(1):269–273.

11.Karimi N, Rezasoltani A, Rahnama L, Noori-Kochi F, Jaberzadeh S. Ultrasonographic analysis of dorsal neck muscles thickness changes induced by isometric contraction of shoulder muscles: a comparison between patients with chronic neck pain and healthy controls. Man Ther. 2016;22: 174–178.

12.Barnsley L,Lord S,Bogduk N：Comparative local anaesthetic blocks in the diagnosis of cervical zygapophysial joint pain.Pain,1993;55:99–106

13.Helgadottir H, Kristjansson E, Mottram S, Karduna A, Jonsson H Jr. Altered scapular orientation during arm elevation in patients with insidious onset neck pain and whiplash-associated disorder. J Orthop Sports Phys Ther. 2010;40(12):784–791.

14.Cagnie B, Struyf F, Cools A, Castelein B, Danneels L, O'Leary S. The relevance of scapular dysfunction in neck pain: a brief commentary. J Orthop Sports Phys Ther. 2014;44(6):435–439.

15.Côté P, van der Velde G, Cassidy JD, et al. The burden and determinants of neck pain in workers: results of the Bone and Joint Decade 2000-2010 Task Force on Neck Pain and Its Associated Disorders. Spine (Phila Pa 1976). 2008;33(4 Suppl):S60-S74

16.Porta M. A comparative trial of botulinum toxin type A and methylprednisolone for The treatment of myofascial pain syndrome and pain from chronic muscle spasm. Pain. 2000;85(1-2):101-105.

17.Gilron I , Jensen TS , Dickenson AH. Combination pharmacotherapy for management of chronic pain: from bench to bedside. Lancet Neurology. 2013;12(11):1084-1095.

18.Graham DY. Prevention of gastroduodenal injury induced by chronic nonsteroidal antiinflamatory drug therapy.Gastroenterology.1989;96(2Pt2 Suppl):675-681.

19.Harirforoosh S, Asghar W, Jamali F. Adverse effects of nonsteroidal antiinflammatory drugs: an update of gastrointestinal, cardiovascular and renal complications. J Pharm Pharm Sci. 2013;16(5):821-847.

20.Parikh P, Santaguida P,Macdermid J,et al. Comparison of CPG's for thediagnosis,prognosis and management of non-specific neck pain：a systematic review. BMC Musculoskelet Disord. 2019;20(1):81.

21.Cheng YH, Huang GC. Effificacy of massage therapy on pain and dysfunction in patients with neck pain: A systematic review and meta-analysis.Evid Based Complement Alternat Med.2014;204360.

22.Pach D , Piper M , Lotz F , et al. Effectiveness and Cost-Effectiveness of Tuina for Chronic Neck Pain: A Randomized Controlled Trial Comparing Tuina with a No-Intervention Waiting List. J Altern Complement Med. 2018;24(3):231-237.

23.Yuan QL, Guo TM, Liu L, et al. Traditional Chinese medicine for neck pain and low back pain: A systematic review and meta-analysis. PLoS One 2015;10(2):e0117146

24.Patel KC, Gross A, Graham N, Goldsmith CH, Ezzo J, Morien A, et al. Massage for mechanical neck disorders. Cochrane Database Syst Rev. 2012;9:CD004871.

25.Kong LJ, Zhan HS, Cheng YW, Yuan WA, Chen B, Fang M. Massage therapy for neck and shoulder pain: a systematic review and meta-analysis. Evid Based Complement Alternat Med. 2013;10(2013):613279.

26.G. C. Goats, "Massage—the scientific basis of an ancient art: part 2—physiological and therapeutic effects," British Journal of Sports Medicine, vol. 28, no. 3, pp. 153–156, 1994.

27.Wang X , Pi Y , Chen B , et al. Effect of traditional Chinese exercise on the quality of life and depression for chronic diseases: a meta-analysis of randomised trials.Sci Rep. 2015;5:15913.

28.Bertozzi L, Gardenghi I, Turoni F, et al. Effect of therapeutic exercise on pain and disability in the management of chronic nonspecific neck pain: systematic review and meta-analysis of randomized trials. Phys Ther 2013; 93(8): 1026–1036.

29.Xiang HP, Liu SJ, Jia HR, et al. A brief analysis on the effect of fitness qigong Yijin method on respiratory muscle[in Chinese]. J Hubei Sports Sci.2006;25:42–46.

30.Zhao LJ, Liu CX, Zhao LB, et al. Effects of Yijinjing exercising combined with Gutong plaster on physiology curvature of cervical spondylosis with cervical type[in Chinese]. Hebei J Trad Chin Med 2017;39:830–834.

31.Liu YJ. The study yijinjing and taijiquan inflfluence of depression on University students [in Chinese]. Sichuan Sport Sci 2013;6:49–51.

32.Hou ZP. Mechanism of yi jinjing for the treatment of sub-health state [in Chinese]. J Liaoing Univ TCM 2009;11:69–70.

33.Yan XH. Effect of yi jinjing on the respiratory health rehabilitation [in Chinese]. J Nangchang Colleg Educ 2010; 25:166.

34.Childs JD, Cleland JA, Elliott JM, et al. Neck pain: Clinical practice guidelines linked to the International Classification of Functioning, Disability, and Health from the Orthopedic Section of the American Physical Therapy Association. J Orthop Sports Phys Ther. 2008;38(9):A1-A34.

35.Goode AP, Freburger J,Carey T. Prevalence, practice patterns, and evidence for chronic neck pain. Arthritis Care Res (Hoboken), 2010, 62(11):1594-1601

36.Skillgate E, Bill AS, Côté P, Viklund P, Peterson A, Holm LW. The effect of massage therapy and/or exercise therapy on subacute or long-lasting neck pain--the Stockholm neck trial (STONE): study protocol for a randomized controlled trial. Trials. 2015; 16:414

37.Bryans R , Decina P , Descarreaux M , et al. Evidence-Based Guidelines for the Chiropractic Treatment of Adults With Neck Pain. J Manipulative Physiol Ther. 2014;37(1):42-63.

38.Wu Z , Kong L , Zhu Q , et al. Efficacy of tuina in patients with chronic neck pain: study protocol for a randomized controlled trial. Trials.2019;20(1):59.

39.Song BL,Fang M. Tuina Therapeutics. Beijing:Chinese Traditional Medicine Publishing House;2016.

40.Yao F. One finger Chan Tuina. Beijing:Chinese Traditional Medicine Publishing House;2018.

41.Lv LJ. Traditional Chinese Exercise of Tuina.Beijing:Chinese Traditional Medicine Publishing House:2016

42.Hui KK, Nixon EE, Vangel MG, Liu J, Marina O, Napadow V, Hodge SM, Rosen BR, Makris N, Kennedy DN: Characterization of the “deqi” response in acupuncture. BMC Complement Altern Med 2007; 7:33.

43.Shi GX, Yang XM, Liu CZ, Wang LP.Factors contributing to therapeutic effects evaluated in acupuncture clinical trials. Trials. 2012; 13:42.

44.Tuttle N. Do changes within a manual therapy treatment session predict between-session changes for patients with cervical spine pain? Aust J Physiother. 2005;51(1):43–48.

45.Bijur PE, Silver W, Gallagher EJ. Reliability of the visual analog scale for measurement of acute pain. Acad Emerg Med. 2001;8(12):1153-1157.

46.Vernon H, Mior S. The neck disability index: a study of reliability and validity. J Manipulative Physiol Ther. 1991;14(7):409–415.

47.W.W.K. Zung, Zung self-rating depression scale and depression status inventory, in: Assessment of Depression, Springer Berlin Heidelberg, Berlin, Heidelberg, 1986, pp. 221-231.

48.Zheng Z , Wang J , Gao Q , et al. Therapeutic evaluation of lumbar tender point deep massage for chronic non-specific low back pain.J Tradit Chin Med, 2012,32(4):534-537.

49.Peerada, Damapong, Naowarat, et al. A Randomized Controlled Trial on the Effectiveness of Court-Type Traditional Thai Massage versus Amitriptyline in Patients with Chronic Tension-Type Headache. Evid Based Complement Alternat Med. 2015;2015:930175.

50.Lluch E, Schomacher J, Gizzi L, Petzke F, Seegar D, Falla D. Immediate effects of active cranio-cervical flflexion exercise versus passive mobilization of the upper cervical spine on pain and performance on the cranio-cervical flflexion test. Man Ther 2014;19(1):25-31.

51.Martel J, Dugas C, Dubois JD, et al.A randomised controlled trial of preventive spinal manipulation with and without a home exercise program for patients with chronic neck pain. BMC Musculoskelet Disord. 2011;12: 41.

52.Edwards J, Knowles N. Superficial dry needling and active stretching in the treatment of myofascial pain--a randomised controlled trial. Acupunct Med.2003;21(3):80-86.

53.Liang BJ,Zhang LC. Effect of cervical spine exercises combined with massage on the treatment of NCNP[in Chinese]. China Rural Medicine. 2016;23(2):41-42.

54.Madson TJ, Cieslak KR, Gay RE. Joint mobilization vs massage for chronic mechanical neck pain: A pilot study to assess recruitment strategies and estimate outcome measure variability. J Manipulative Physiol Ther. 2010;33(9):644-651.

55.Schwerla F, Bischoff A, Nurnberger A, Genter P, Guillaume JP, Resch KL. Osteopathic treatment of patients with chronic non-specifific neck pain: A randomised controlled trial of efficacy. Forsch Komplementmed. 2008;15(3):138-145.

56.Zhang Q, Wang YG, Liu K. Research progresses on application of Yi Jin Jing (Sinew-transforming Qigong Exercises)[in Chinese]. Henan Zhongyi, 2013, 33(5): 774-776.

57.Guo CQ, Cheng YW, Kong LJ, Bu JH, Yang XC．On Gongfa Gongli of Yi Jin Jing from the perspective of biomechanics[in Chinese]. Changchun Zhongyiyao Daxue Xuebao, 2014, 30(2): 262-264.

58.Hu WM, Gong L. The preliminary experience of spreading tuina Yijinjing Gong in the community[in Chinese]. Anmo Yu Kangfu Yixue, 2012, 2(10): 2-3.

59.Zhang WC, Zhong ZB, Wu QH, Chen XF, Wang SY, Zhang Y. A research on health Qigong-Yi Jin Jing for slowing down intelligence decline of the aged[in Chinese]. Zhongguo Xingwei Yixue Kexue, 2006, 15(9): 827.
