# Supplementary material for: Effectiveness of Tuina Therapy Combined With Yijinjing Exercise in the Treatment of Nonspecific Chronic Neck Pain: A Randomized Clinical Trial
Source: JAMA Netw Open. 2022 Dec 13;5(12):e2246538. doi: 10.1001/jamanetworkopen.2022.46538 (PMC9856335; doi:10.1001/jamanetworkopen.2022.46538)
Supplement: Supplement 2. — eAppendix 1. Tuina Protocol eAppendix 2. Yijinjing Protocol eAppendix 3. Correlation Analysis [file jamanetwopen-e2246538-s002.pdf]

Supplementary Online Content

Cheng Zj, Zhang Sp, Gu Yj, et al. Effectiveness of tuina therapy combined with yijinjing exercise in the treatment of nonspecific chronic neck pain: a randomized clinical trial. *JAMA Netw Open*. 2022;5(12):e2246538. doi:10.1001/jamanetworkopen.2022.46538

eAppendix 1. Tuina Protocol
eAppendix 2. Yijinjing Protocol
eAppendix 3. Correlation Analysis

This supplementary material has been provided by the authors to give readers additional information about their work.

© 2022 Cheng Z et al. *JAMA Network Open.*

eAppendix 1. Tuina Protocol

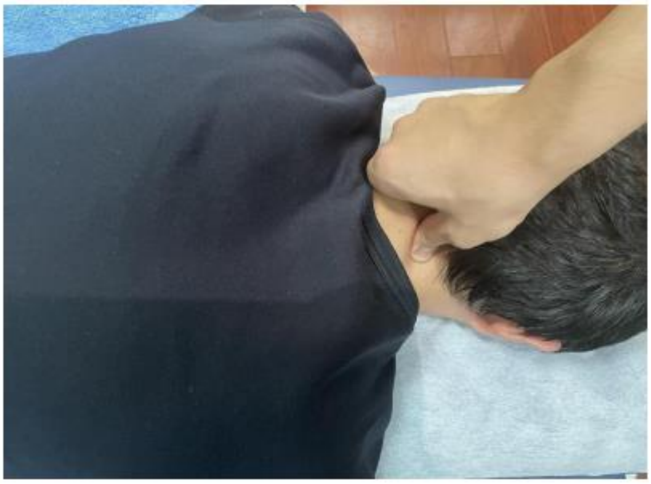

Step one: soft tissue manipulation

The patient's symptoms were assessed in detail before each treatment. With a prone position, they were told to relax naturally initially. Pressing-kneading manipulation was used to relax soft tissue and stiff muscles of neck and shoulder by the therapist for 5min. The purpose of this step is to resolve adhesion and increase blood circulation.

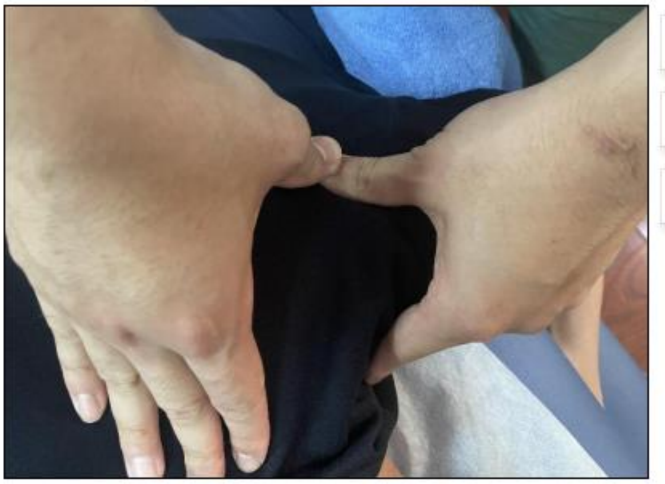

Step two:clicking acupoint manipulation

GB20, DU16,GB21,SJ14 and SI14 were chosen with pressing-kneading manipulation for 2 min respectively.

© 2022 Cheng Z et al. *JAMA Network Open.*

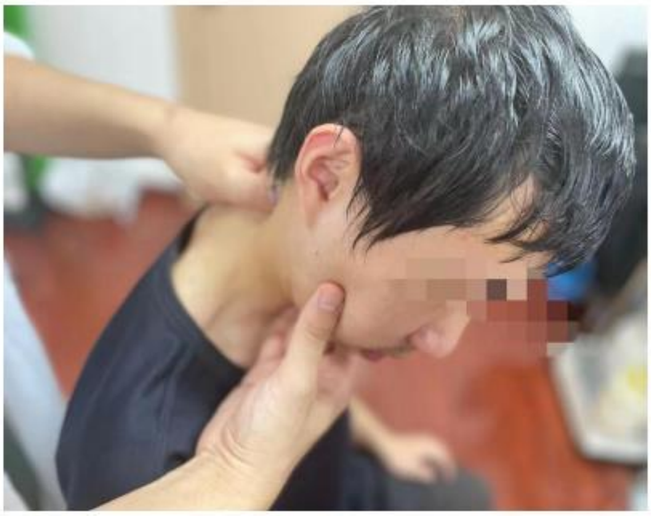

Step three: spinal manipulation

The patients were told to sit in orthopnea position for the safety of this spinal manipulation. First, shaking method was used to relax the soft tissue of neck and shoulder. Second, deviated spinous process was pressed by thumb and with another hand to hold the lower jaw for tracing and twisting the neck. Finally, an abrupt pulling motion was made to advance the stretch by 5 to 10 degrees.

© 2022 Cheng Z et al. *JAMA Network Open.*

eAppendix 2. Yijinjing Protocol

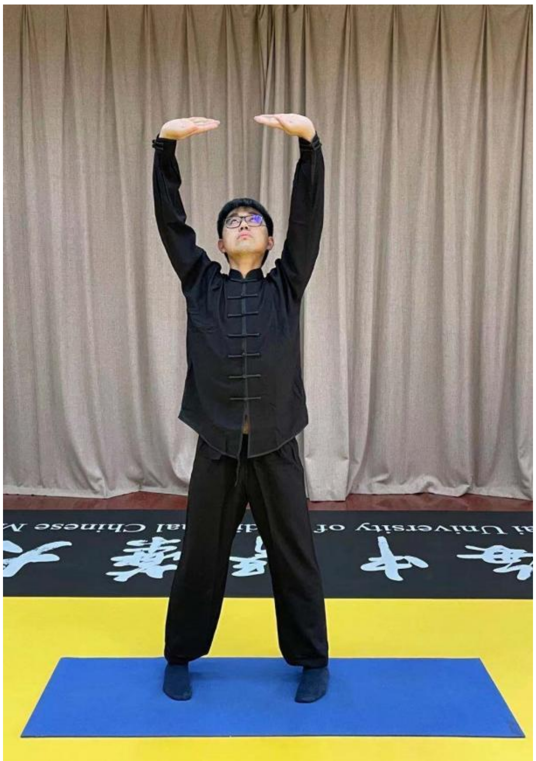

Step one:The Third Aspect of Wei-tuo

Take a step to the left, raise hands in front of chest with palms up,fingertip facing. Rotate wrists and raise the palms above the head. Slightly bend the elbow, look upward towards the dorsum of the palm. Lift the heels, stand on toes. Hold for 5 seconds. Then make fists, rotate the wrists and put the fists down to the waist slowly. Place whole feet on ground. Repeat the whole procedure 3 times in 6 min.

© 2022 Cheng Z et al. *JAMA Network Open.*

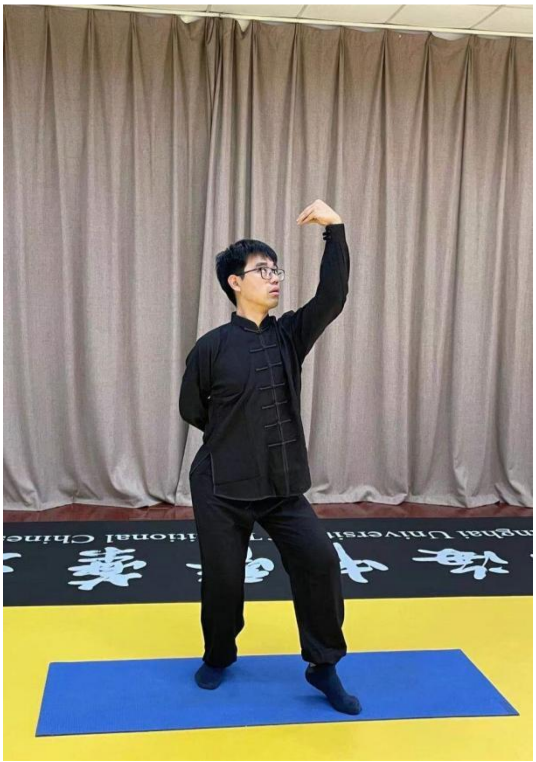

Step two:Taking away a star and changing the Dipper for it

Move right foot to right front. Raise and straighten the right hand, direct the palms facing downward, slightly turn the head to the right side and fix the eyes on the right palm. Bend the left elbow and put it naturally on the back of the body. Hold for 5 seconds. Then put hands to both sides of body and relax when exhaling Exchange the left side for exercising. Repeat the whole procedure 3 times in 6 min.

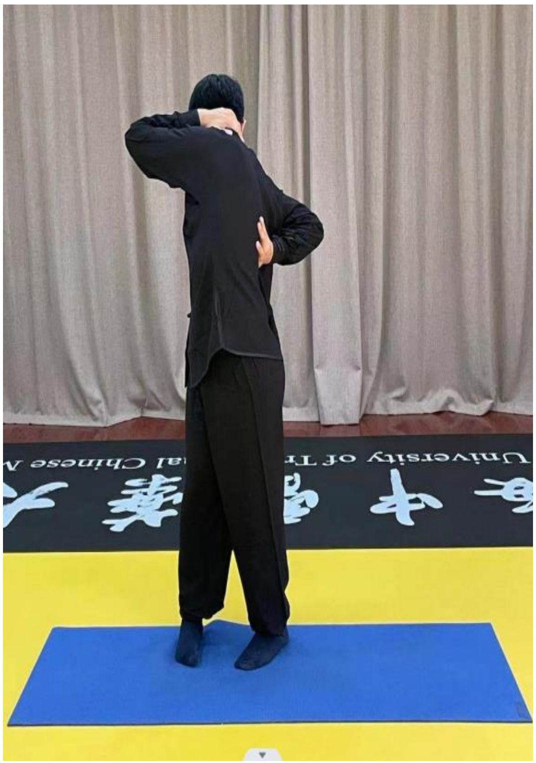

Step three: Nine demons drawing their swords

Take a step to the left. Cross hands over chest and raise up. Separate hands above head. Put left hand on the neck and right hand to the back .Rise head to the left 45°and twist waist. Pull hands tightly when exhaling. Hold for five seconds. Then put hands to both sides of body and relax when exhaling. Exchange to right side for exercising. Repeat the whole procedure 3 times in 6 min.

© 2022 Cheng Z et al. *JAMA Network Open.*

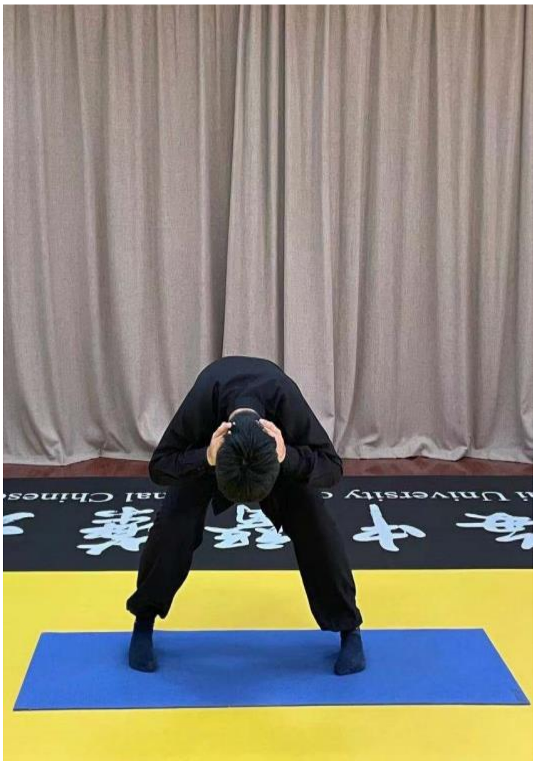

Step four:Bowing in salutation

Separate the legs and hold the head by the hands. Bend the waist to between the knees. Stretch the head to between the legs. Use finger to tap the head for 7 times. Then straighten the knee and waist when exhaling and stand upright. Repeat the whole procedure 3 times in 6 min.

© 2022 Cheng Z et al. *JAMA Network Open.*

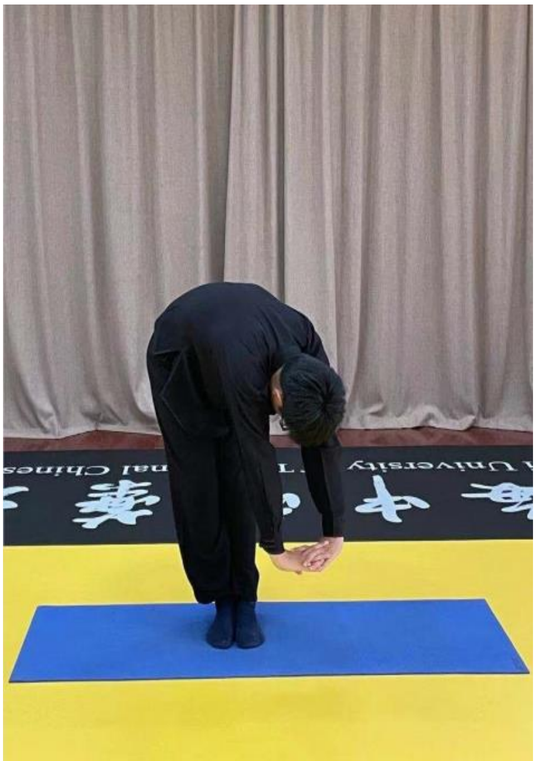

Step five: Wagging the Tail

Take a step to the left. Cross hands in front of chest and raise hands above head. Fix eyes to hands.Then bend the upper body upward and press down your hands to the ground .Raise up head and open eyes. Bend body and place heels when exhaling . Strengthen body and lift heels when inhaling. Repeat the whole procedure 3 times in 6 min. Patient can adjust the degree of flexion and extension according his own physical condition.

© 2022 Cheng Z et al. *JAMA Network Open.*

eAppendix 3. Correlation Analysis

Correlation analysis was conducted among the difference of the tissue hardness of left and right upper trapius muscle and the difference of scales and AROM before(week 0)and after intervention(week 8) in Yijinjing combined with Tuina Group.

A. The correlation between the difference in the left upper trapezius muscle and the difference in extension in Yijinjing combined with Tuina Group

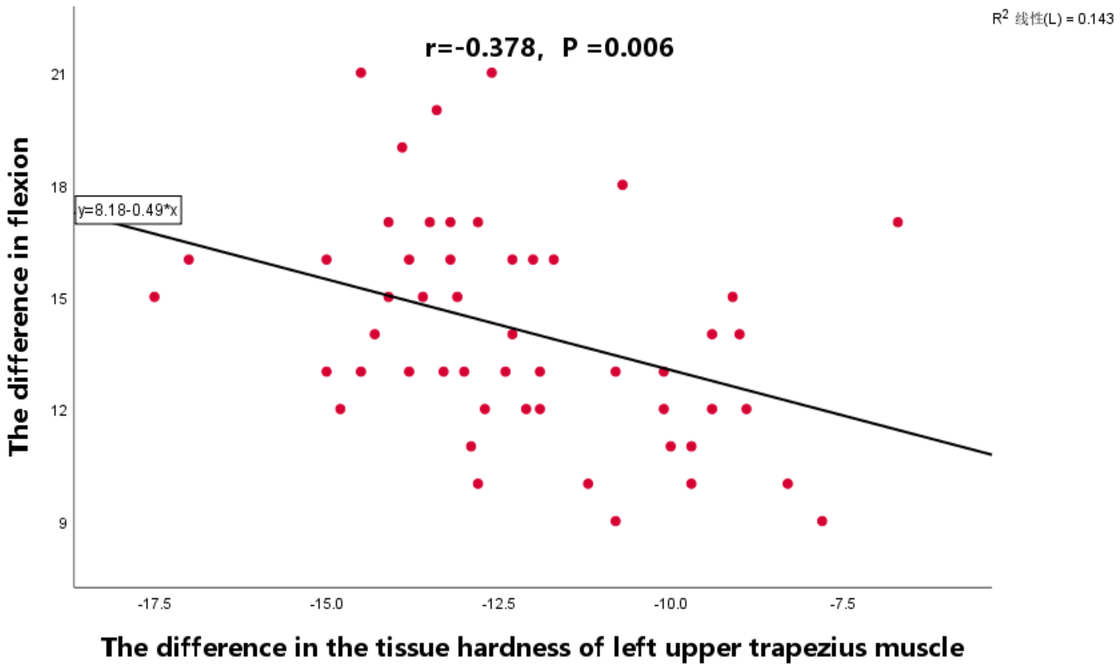

The difference in the tissue hardness of left upper trapezius muscle was negatively correlated with the difference in flexion (r=-0.378,*P*=0.006).

© 2022 Cheng Z et al. *JAMA Network Open.*

B. The correlation between the difference in the left upper trapezius muscle and the difference in extension in Yijinjing combined with Tuina Group

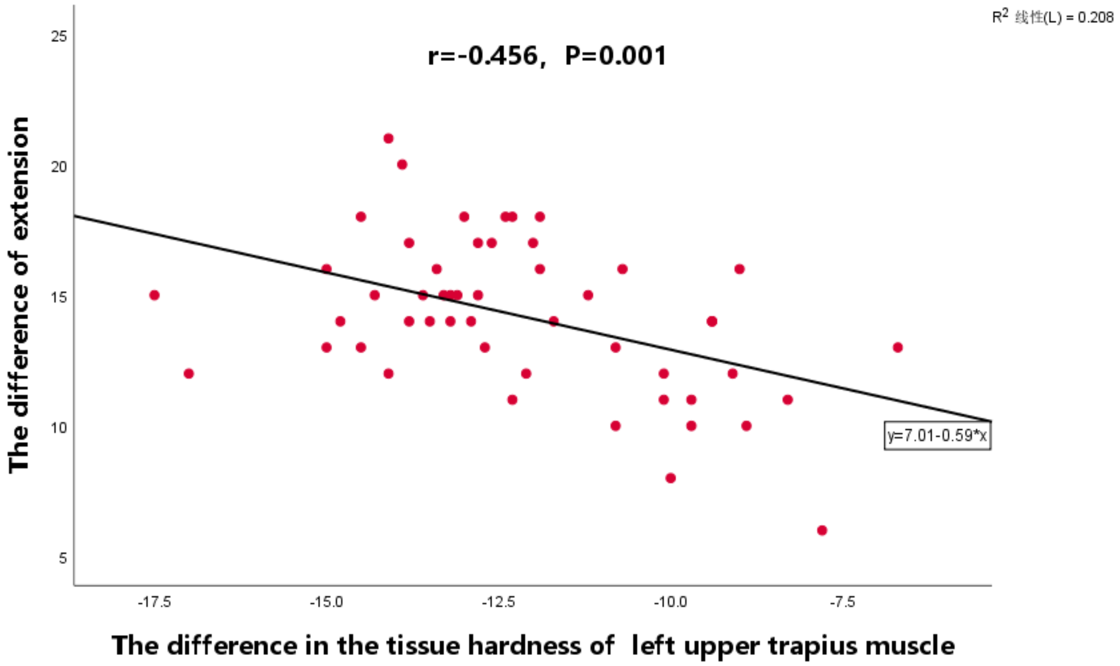

The difference in the tissue hardness of the left upper trapezius muscle was negatively correlated with the difference in extension (r=-0.456, *P*=0.001)

© 2022 Cheng Z et al. *JAMA Network Open.*

C. The correlation between the difference in the left upper trapezius muscle and the difference in right rotation in Yijinjing combined with Tuina Group

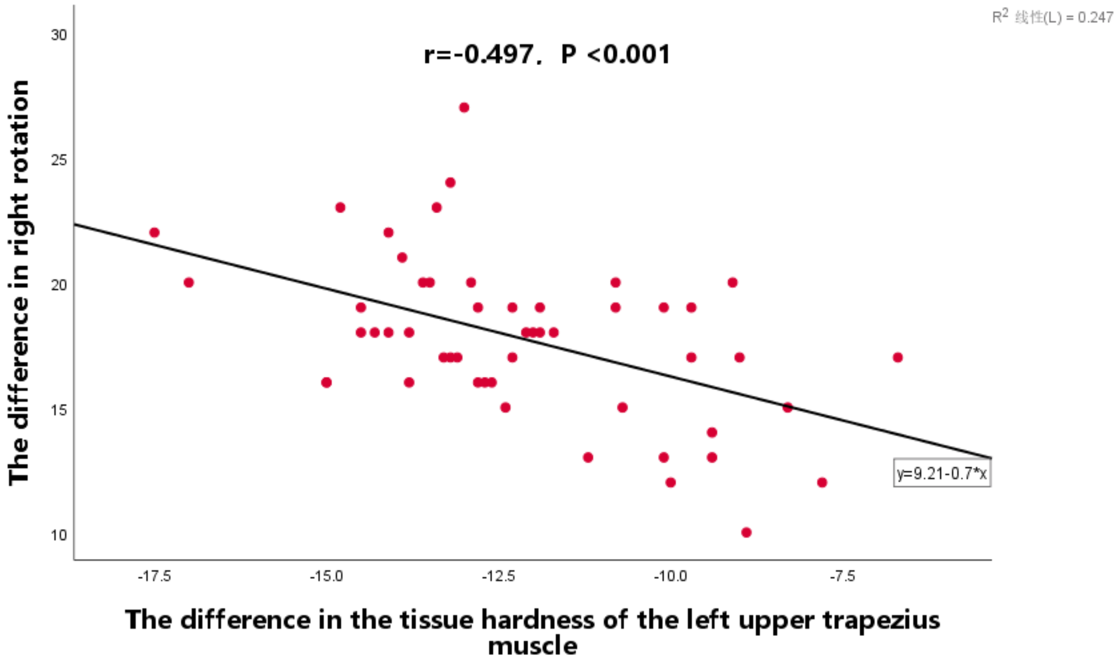

The difference in the tissue hardness of the left upper trapezius muscle was negatively correlated with the difference in right rotation (r=-0.497, P<0.001).

D. The correlation between the difference in the left upper trapezius muscle and]the difference in the right upper trapezius muscle in Yijinjing combined with Tuina Group

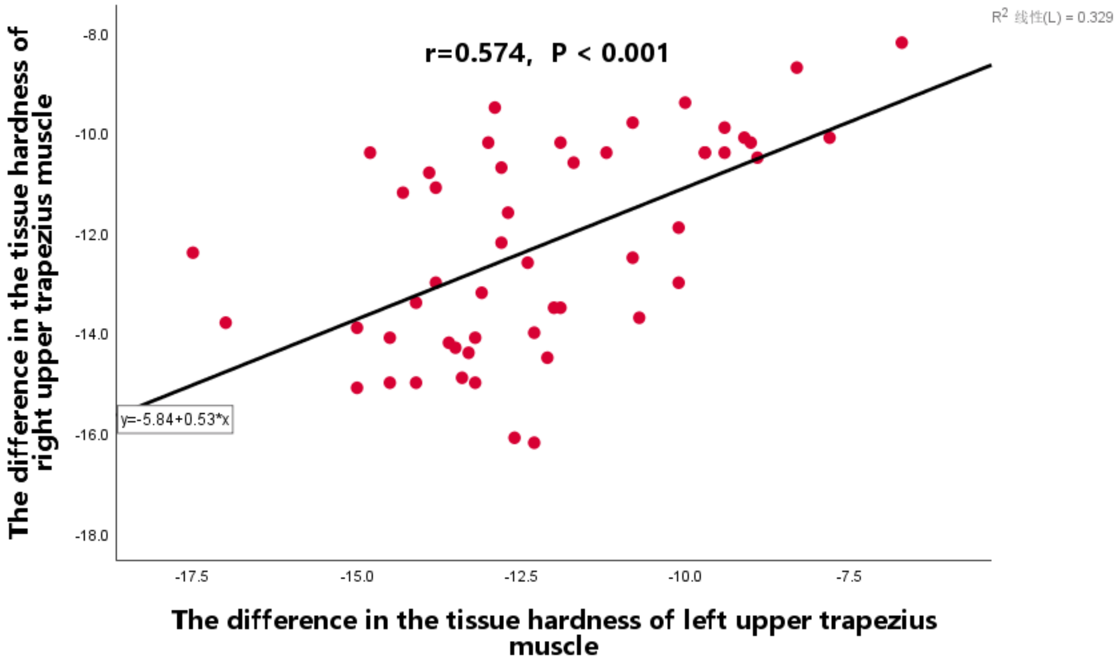

The difference in the tissue hardness of left upper trapezius muscle was positively correlated with the difference in the tissue hardness of right upper trapezius muscle (r=0.574, *P* <0.001).

© 2022 Cheng Z et al. *JAMA Network Open.*

E. The correlation between the difference in the right upper trapezius muscle and the difference in flexion in Yijinjing combined with Tuina Group

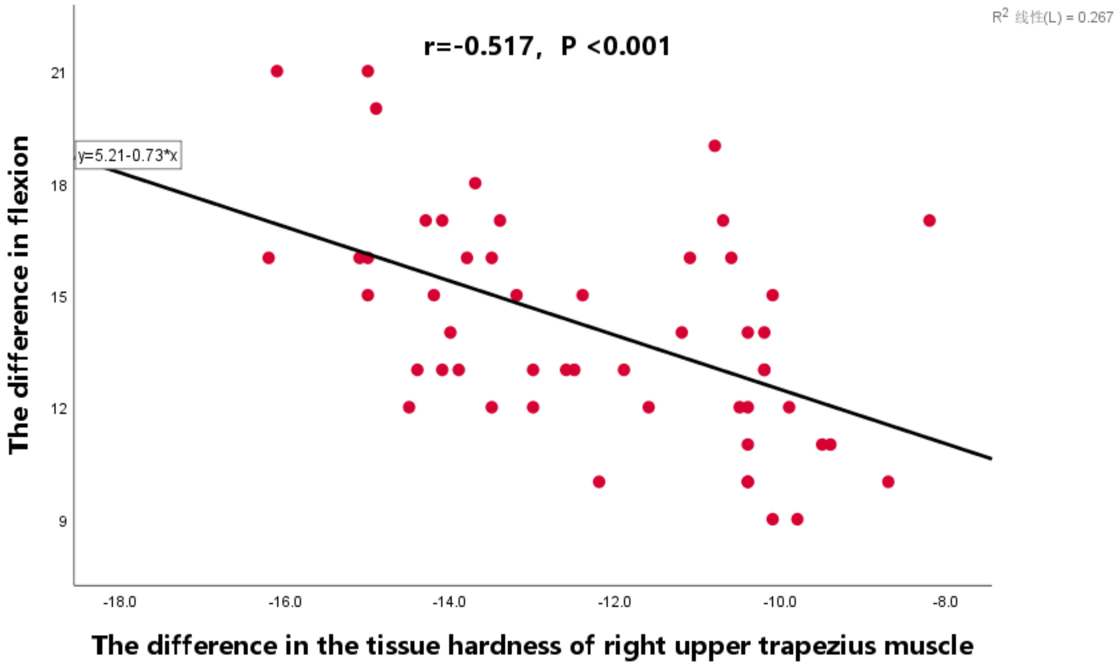

The difference in the tissue hardness of right upper trapezius muscle was negatively correlated with the difference in flexion (r=-0.517, *P* <0.001).

© 2022 Cheng Z et al. *JAMA Network Open.*

F. The correlation between the difference in the right upper trapezius muscle and the difference in extension in Yijinjing combined with Tuina Group

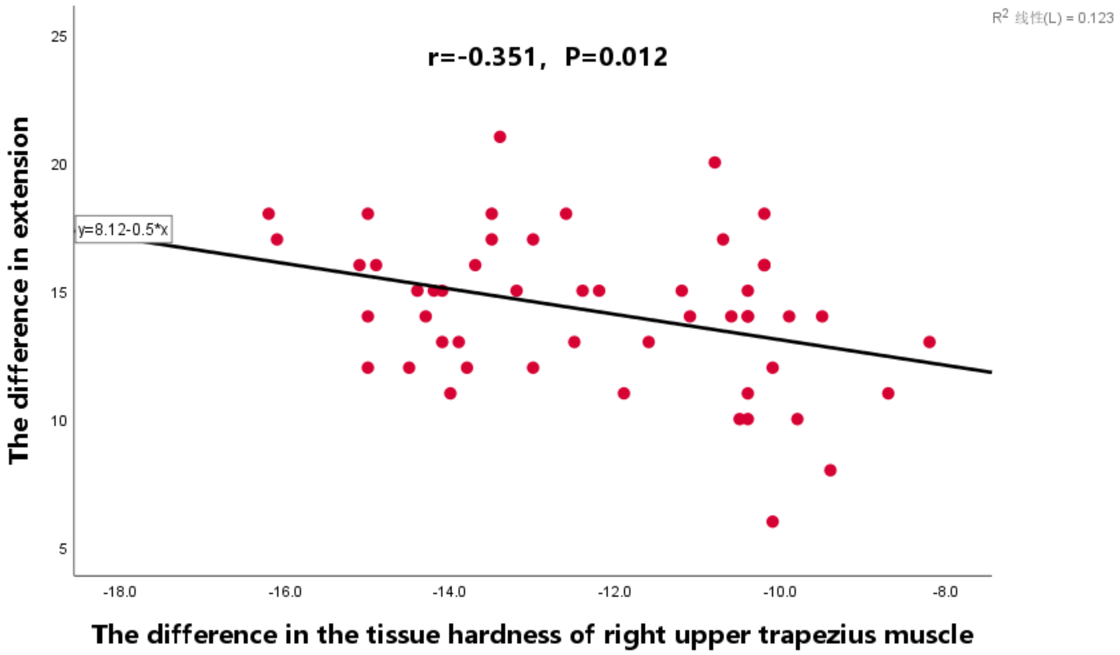

The difference in the tissue hardness of right upper trapezius muscle was negatively correlated with the difference in extension(r=-0.315,*P* <0.001).

© 2022 Cheng Z et al. *JAMA Network Open.*

G. The correlation between the difference in the right upper trapezius muscle and the difference in SAS in Yijinjing combined with Tuina Group

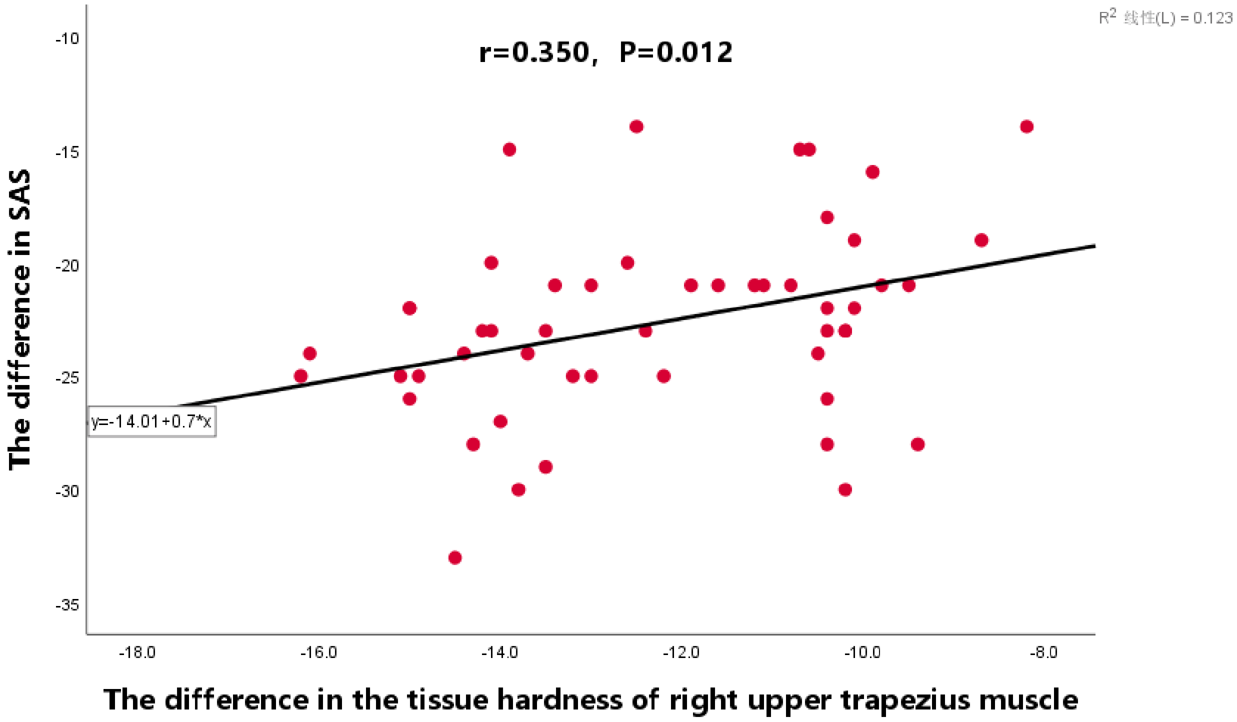

The difference in the tissue hardness of right upper trapezius muscle was positively correlated with the difference in SAS score (r=0.350, *P*=0.012).

© 2022 Cheng Z et al. *JAMA Network Open.*
