# Supplementary material for: Effectiveness of Tuina Therapy Combined With Yijinjing Exercise in the Treatment of Nonspecific Chronic Neck Pain: A Randomized Clinical Trial
Source: JAMA Netw Open. 2022 Dec 13;5(12):e2246538. doi: 10.1001/jamanetworkopen.2022.46538 (PMC9856335; doi:10.1001/jamanetworkopen.2022.46538)
Supplement: Supplement 3. — Data Sharing Statement [file jamanetwopen-e2246538-s003.pdf]

Data Sharing Statement

Cheng. Effectiveness of Tuina Therapy Combined With Yijinjing Exercise in the Treatment of Nonspecific Chronic Neck Pain. *JAMA Netw Open*. Published December 13, 2022. doi:10.1001/jamanetworkopen.2022.46538

Data
Data available: Yes
Data types: Deidentified participant data
How to access data: Send request email to Professor Yao, doctoryaofei@126.com.
When available: With publication

Supporting Documents
Document types: None

Additional Information
Who can access the data: Researchers whose proposed use of the data has been approved
Types of analyses: For a specified purpose
Mechanisms of data availability: After approval of a proposal, and with a signed data access agreement
